# Expression of the Maize *Dof1* Transcription Factor in Wheat and Sorghum

**DOI:** 10.3389/fpls.2017.00434

**Published:** 2017-03-30

**Authors:** Pamela A. Peña, Truyen Quach, Shirley Sato, Zhengxiang Ge, Natalya Nersesian, Taity Changa, Ismail Dweikat, Madhavan Soundararajan, Tom E. Clemente

**Affiliations:** ^1^Department of Agronomy and Horticulture, University of Nebraska-LincolnLincoln, NE, USA; ^2^Center for Biotechnology, University of Nebraska-LincolnLincoln, NE, USA; ^3^Department of Biochemistry, University of Nebraska-LincolnLincoln, NE, USA; ^4^Center for Plant Science Innovation, University of Nebraska-LincolnLincoln, NE, USA

**Keywords:** abiotic stress, nitrogen use efficiency, transcription factor, wheat, sorghum

## Abstract

Nitrogen is essential for plant growth and development. Improving the ability of plants to acquire and assimilate nitrogen more efficiently is a key agronomic parameter that will augment sustainability in agriculture. A transcription factor approach was pursued to address improvement of nitrogen use efficiency in two major commodity crops. To this end, the *Zea mays* Dof1 (*ZmDof1*) transcription factor was expressed in both wheat (*Triticum aestivum*) and sorghum (*Sorghum bicolor*) either constitutively, UBI4 promoter from sugarcane, or in a tissue specific fashion via the maize rbcS1 promoter. The primary transcription activation target of *ZmDof1*, phospho*enol*pyruvate carboxylase (PEPC), is observed in transgenic wheat events. Expression *ZmDof1* under control of the rbcs1 promoter translates to increase in biomass and yield components in wheat. However, constitutive expression of *ZmDof1* led to the down-regulation of genes involved in photosynthesis and the functional apparatus of chloroplasts, and an outcome that negatively impacts photosynthesis, height, and biomass in wheat. Similar patterns were also observed in sorghum transgenic events harboring the constitutive expression cassette of *ZmDof1*. These results indicate that transcription factor strategies to boost agronomic phenotypic outcomes in crops need to consider expression patterns of the genetic elements to be introduced.

## Introduction

Aside from environment, crop yields are influenced by two factors, the underlying genetics of the seed sown and the agronomic inputs practiced. In regards to the latter, nitrogen is a key nutrient and is essential for plant growth and development. During the past five decades, cereal crops such as wheat and sorghum have relied on nitrogen-based fertilizers as an agronomic practice to maximize yield and plant productivity (Good et al., 2004). The world's population is expected to increase substantially by 2,050, with estimates of 9 billion. Nitrogen fertilizer use will rise by at least 3-fold to ensure food security for such a population size (Food and Agriculture Organization of the United Nations, 2009). Given the increasing concerns regarding the detrimental effect of excessive nitrogen applications on the environment and its impact on production costs, strategies to enhance crop genetics to improve nitrogen use efficiency (NUE) have become essential targets in the plant sciences (Zeigler and Mohanty, 2010). To address this goal, gaining insight on the genetic underpinnings that govern modulation of nitrogen (N) uptake, assimilation, and remobilization in plants is essential.

Nitrogen use efficiency (NUE) is a complex trait that involves multiple interacting genetic and environmental factors. A number of transgenic approaches have been explored to enhance NUE in plants through the overexpression or knockout mutations of genes involved in nitrogen uptake, nitrate reduction, N assimilation, remobilization, recycling, amino acid biosynthesis, regulation of carbon/nitrogen metabolism, and signaling (Good et al., 2004; McAllister et al., 2012; Xu et al., 2012). Ectopic expression of *Arabidopsis* nitrogen transporter *AtNRT1*.1 gene resulted in increased nitrate uptake in *Arabidopsis* (Liu et al., 1999), and the ammonium transporter, *OsAMT1-1*, in rice enhanced ammonium uptake, which led to the increase in content of chlorophyll, starch, sugars, and grain yield in transgenic rice (Bao et al., 2015). In contrast, overexpression of the *OsAMT1-3* induced carbon/nitrogen imbalances in transgenic rice, resulting in poor growth, and low yield (Bao et al., 2015). Moreover, manipulation of glutamine synthetase (GS)/glutamate synthase (GOGAT) cycle genes have been shown to enhance growth rate, yield, and biomass in tobacco, poplar, wheat, rice, and maize (Habash et al., 2001; Martin et al., 2006; Cai et al., 2009; Brauer et al., 2011; Molina-Rueda and Kirby, 2015; Seger et al., 2015). Genetic perturbation of the GS/GOGAT cycle as a means to enhance NUE can translate to different phenotypic outcomes depending on the environment. For example, ectopic expression of *Os*GS1 in rice led to increased accumulation of total nitrogen under low and normal nitrogen conditions in plants grown hydroponically or in controlled growth chambers. In contrast, yield was reduced up to 33% when plants were grown under field conditions (Cai et al., 2009). In tobacco, expression of *Ps*GS1 and *Ms*GS1 improved growth and total dry weight under controlled low nitrogen conditions (Fuentes et al., 2001; Oliveira et al., 2002), however, translation to seed yield is not agronomic parameter of importance to this crop. In addition to genetic approaches to modulate GS/GOGAT cycle, genes involved in amino acid biosynthesis such as alanine aminotransferase (AlaAT), asparagine synthease (AS), aspartate aminotransferase (AspAT), and glutamate dehydrogenase (GDH) have also been overexpressed as a means to impart NUE in plants, including rice, rapeseed, lettuce, *Arabidopsis*, tobacco, and tomato (Giannino et al., 2007; Good et al., 2007; McAllister et al., 2012). AlaAT for instance, has been shown to maintain the carbon-nitrogen balance in plants through the translocation of pyruvate or alanine. *Arabidopsis* and rice plants overexpressing an AlaAT from barley exhibited enhanced biomass and seed yield under low nitrogen conditions (Good et al., 2007; Shrawat et al., 2008).

In addition to these single gene strategies to enhance NUE, the use of transcription factors as a route to modulate multiple genes in a metabolic pathway has also been explored (Century et al., 2008). To this end the maize zinc finger protein Dof1 (Yanagisawa et al., 2004), belonging to a family known as DOF (DNA binding with one finger) have been investigated. Members of the DOF family are present in a wide range of organisms including *Chlamydomonas reindhardtii, Physcomitrella patens*, gymnosperms, and all angiosperms. *In silico* analyses have led to the identification of 37 putative *Dof* genes in *Arabidopsis thaliana*, 54 in *Zea mays*, 30 in *Oryza sativa*, 36 in *Sorghum bicolor*, 24 in *Hordeum vulgare*, 31 in *Triticum aestivum*, 37 in *Solanum lycopersicon*, 41 in *Populus trichocarpa* and 78 in *Glycine max* (Lijavetzky et al., 2003; Yang et al., 2006; Shaw et al., 2009; Kushwaha et al., 2011; Guo and Qiu, 2013). These Dof proteins have been associated with regulation of genes involved in vascular development, light signaling, carbohydrate metabolism, phloem sugar transport, photosynthetic carbon assimilation, flowering time, dormancy, response to phytohormones, storage protein synthesis, and phytochrome signaling (Noguero et al., 2013).

Nitrogen assimilation is intimately linked with carbon metabolism. For example, nitrogen allocation toward regeneration of Rubiso and light harvesting complexes are essential for photosynthesis (Zhu et al., 2008). In turn, photosynthesis plays a central role in nitrogen metabolism by providing ATP, carbon skeletons, and reducing agent required for assimilation of the nutrient (Funk et al., 2013). Hence, avenues to modulate carbon/nitrogen (C/N) networks hold promise as a strategy to enhance NUE in plants. The Dof1 from maize (*ZmDof1*) has been shown to upregulate the expression of the C_4_-phosphoenol pyruvate carboxylase (PEPC), the initial carbon fixing enzyme of C_4_ species and a key component of the TCA cycle. *ZmDof2* acts as a repressor of PEPC transcription by blocking the transactivation by Dof1 (Yanagisawa, 2000). The expression of *ZmDof1* in Arabidopsis and potato was shown to modulate C/N network, promoting nitrogen assimilation and increasing plant growth under low nitrogen conditions (Yanagisawa et al., 2004). Transient assays in leaf protoplasts showed the transactivation of rice PEPC promoter elements, by *ZmDof1*, while stable expression revealed an increased PEPC expression and modulation of metabolites associated with the TCA cycle. Moreover, rice *ZmDof1* transgenic events displayed photosynthesis rate and total nitrogen and carbon boosts under low nitrogen conditions (Kurai et al., 2011). However, in contrast with the positive agronomic phenotypes shown in rice, potato, and Arabidopsis, an attempt to translate these agronomic outcomes to *Populus* was not successful (Lin et al., 2013).

To elucidate the effects of modulating C/N networks and its impact on NUE in both wheat and sorghum, we introduced the *ZmDof1*, under the control of two regulatory elements, a constitutive version governed by sugarcane UBI4 promoter (Wei et al., 2003) and a tissue specific cassette regulated maize *rbc*S1 promoter (Sattarzadeh et al., 2010). The derived transgenic wheat events were characterized at the molecular level and phenotyped under both controlled and field environments, while the sorghum events were phenotyped under greenhouse conditions.

## Materials and methods

### Vector construction

**Figure 1:**
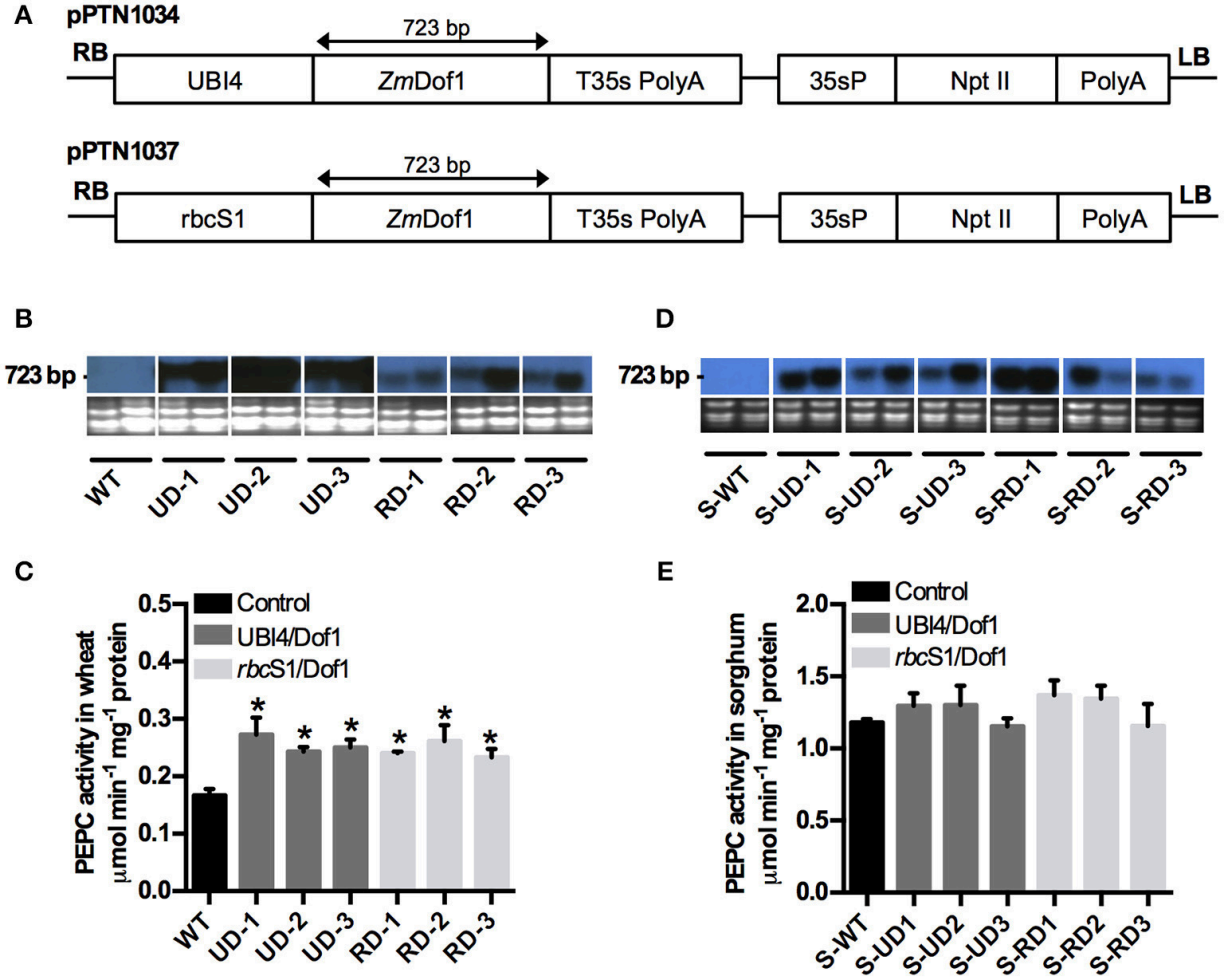
**Production of transgenic events expressing *ZmDof1* from maize. (A)** Diagram of the expression cassette pPTN1034 (UBI4/Dof1) harboring a coding region of the Dof1 transcription factor from maize under the control of the UBI4 promoter, and a pPTN1037 (*rbc*S1/Dof1) expression cassette containing the Dof1 coding region under the maize *rbc*S1 promoter; both expression cassettes contained a 35s poly A terminator sequence for the Dof1 coding region and the nptII gene for plant selection. RB, right border; UBI4, sugarcane (*Saccharum officinarum*) ubiquitin promoter ^**^; rbcS1, *Zea mays* Rubisco subunit 1 promoter; *Zm*Dof1, *Z*. *mays* Dof1 transcription factor; T35s PolyA, CaMV terminator 35s poly A; 35sP, CaMV 35s promoter; nptII, neomycin phosphotransferase II; Poly A, CaMV terminator 35s poly A; LB, left border. **(B)** Expression analysis of the Dof1 transcription factor in leaf tissue of wheat control plants (WT)-black bars; three UBI4/Dof1 wheat transgenic events (UD-1, UD-2, UD-3)-gray bars, and three *rbc*S1/Dof1 wheat transgenic events (RD-1, RD-2, RD-3)-silver bars. RNA on bottom showing equal loading. The northern blot was hybridized with a fragment of the *Zm*Dof1 gene (504 bp). **(C)** PEPC activity from wheat plants measured spectrophotometrically using enzyme extracts from leaves at anthesis. **(D)** Expression analysis of the Dof1 transcription factor in leaf tissue of sorghum control plants (S-WT)-black bars; three UBI4/Dof1 sorghum transgenic events (S-UD-1, S-UD-2, S-UD-3)-gray bars, and three *rbc*S1/Dof1 sorghum transgenic events (S-RD-1, S-RD-2, S-RD-3)-silver bars. RNA on bottom showing equal loading. The northern blot was hybridized with a fragment of the *Zm*Dof1 gene (504 bp). **(E)** PEPC activity from sorghum plants measured spectrophotometrically using enzyme extracts from leaves at anthesis. Data expressed as mean ± *SE* (*n* = 3). Asterisks indicate significant differences from the control (*p* < 0.05).

A cDNA (*Zm*Dof1, 723 bp) for maize (*Zea mays*) transcription factor Dof1 was synthesized and codon optimized (GenScript, Piscataway, NJ, USA) based on the GenBank accession NP001105709.1. Two binary plasmids referred to as pPTN1034 and pPTN1037 were designed to harbor *ZmDof1* under the control of a constitutive UBI4 promoter from sugarcane (Wei et al., 2003) and a tissue specific regulated via the maize *rbc*S1 promoter (Sattarzadeh et al., 2010), respectively (Figure 1A). The *ZmDof1* cDNA was subcloned downstream of each promoter and terminated with the T35s polyadenylation signal. Each plant expression cassette was cloned into the binary plasmid pPZP212 (Hajdukiewicz et al., 1994), that carries a neomycin phosphotrasnferase II (NPTII) cassette for plant selection.

### Plant transformation

The binary vectors were mobilized into *Agrobacterium tumefaciens* strain C58C1/pMP90 (Koncz and Schell, 1986) and NTL_4_/pKPSF2 (Luo et al., 2001) via tri-parental mating, the resultant transconjugants were utilized for wheat (*Triticum aestivum*) and sorghum (*Sorghum bicolor)* transformation, respectively. Wheat transformations were conducted with a spring wheat genotype CB037 as previously described (Clemente and Mitra, 2004). Sorghum transformation were conducted with the grain genotype, TX430, following the protocol outlined by Howe et al. (2006).

### Identification of transgenic plants

The identification of transformed T_0_ and T_1_ plants was made by monitoring the expression of *nptII* via ELISA (Agdia® Cat# PSP73000/0480) following manufacturer instructions. Identification of T_2_ and T_3_ plants was done by PCR. Genomic DNA was isolated based on a modified CTAB method (Springer, 2010). Amplification of the synthetic *ZmDof1* was carried out with the primer sets 5′-TGTGTTCAACAGTCAGTTTTTG-3′ and 5′-GGCTGGGAGGTGTTGTAGTTGT-3′ for pPTN1034 and 5′-GTCCTGTCCTGTACTGCGTCCT-3′ and 5′-GGCTGGGAGGTGTTGTAGTTGT-3′ for pPTN1037. The PCR reaction contained ~100 ng DNA, 0.5 μM of each primer, and 1X GoTaq® Green Master Mix. The conditions used for PCR were 95°C for 2 min, 35 cycles of 95°C for 20 s, 56°C (pPTN1034) or 58°C (pPTN1037) for 30 s and 72°C for 1 min, and a final extension of 72°C for 10 min. PCR products were separated by electrophoresis on a 0.8% agarose gel.

### Northern blot analyses of transgenic plants

Gene expression was assayed via northern blot hybridization. RNA was isolated from young leaves using TRIzol® reagent (Invitrogen cat. # 10296-028) following the manufacturer's protocol. RNA quality and concentration was determined with NanoDrop® ND 1000 Spectrophotometer (Thermo Fisher). Fifteen micrograms of RNA were used for northern blot hybridizations. The RNA samples were separated by electrophoresis on 1% agarose gels. The samples were blotted and UV crossed linked to a nylon membrane (Bio-Rad cat #162-0196, Hercules, CA, USA). Membranes were hybridized with dCT^32^P labeled 507 bp region of *ZmDof1* ORF using random prime labeling (Prime-It II Cat # 300385, Stratagene, La Jolla, CA, USA) as previously described (Eckert et al., 2006).

### Phospho*enol*pyruvate carboxylase assay

Flag leaf tissue (50–100 mg) at anthesis was flash frozen in liquid nitrogen and homogenized in an extraction buffer containing 100 mM Tris-HCL pH 7.5, 10 mM MgCl_2_, 14 mM 2-mercaptoethanol, 1 mM EDTA and 5% glycerol (v/v) Soluble protein concentration of the mixture was determined according to Bradford (1976). PEPC activity was measured following the procedures of Maranville and Madhavan (2002), in an assay buffer containing 50 mM Tris-HCl, pH 8.0, 5 mM MgCl_2_, 0.15 mM NADH, 10 mM NaHCO_3_, 2 IU malic dehydrogenase, 4 mM PEP, and the reaction was initiated by adding the extract containing 7 μg of total protein.

### Hydroponic system for wheat

The evaluation of the selected wheat events harboring *ZmDOF1* under controlled hydroponic system was carried out under greenhouse conditions. Wheat seeds were germinated in 1:1 sand: fine vermiculite. Segregation ratio of the transgenic allele within each event was determined via PCR analysis. Lineages carrying the transgenic allele were selected for the study. Two nitrogen levels were evaluated: 15 mM NO_3_ and 0.3 mM NO_3_ in a modified Hoagland solution (Hoagland and Arnon, 1950). Treatments were started 1 week after planting and replaced three times per week over 4 weeks. Leaf chlorophyll levels were monitored using a Minolta SPAD and the meter readings were converted to concentration (μmol/m^2^) using the exponential equation: μmol/*m*^2^ = 10^(*M*0.265)^, *r*^2^ = 0.94 (Markwell et al., 1995). Chlorophyll measurements, expression analysis, and PEPC enzymatic reactions were made in two upper most expanded leaves as previously described. Leaf tissue was fixed in liquid nitrogen and stored at −80°C until the assays were conducted. Fresh and dry biomass was measured upon the collection of the leave tissue.

### Soil-less potting phenotyping system for sorghum

Three independent events per construct were selected. T_2_ generation plants and wild type were germinated and grown under greenhouse conditions in a mixture of 60% vermiculite, 20% sand, and 20% Metro Mix 200. Treatments consisted of modified Hoagland solution containing 15 mM NO_3_ or 0.75 mM NO_3_ (Hoagland and Arnon, 1950). Treatments were applied at watering three times per week. Physiological measurements and enzymatic analysis were made at anthesis. Leaves used for chlorophyll measurement were also used to determine PEPC activity as described above. Leaves where flash frozen in liquid nitrogen and stored at –80°C until the assays were conducted. Fresh and dry biomass was measured upon the collection of the leave tissue.

Greenhouse ambient light was supplemented with a combination of metal halide and high pressure sodium light fixtures. Photoperiods of 15 and 10 h were utilized for greenhouse wheat and sorghum experiments, respectively. The greenhouse environment provides instantaneous light intensities ranging between 600 and 800 μmol m^−2^ s^−1^ under cloudy conditions, while under full sun, intensities can reach between 1,100 and 1,300 μmol m^−2^ s^−1^.

### Field phenotyping

Field trials were conducted on two independent events containing UBI4/*ZmDof1* and two containing rbcS1/*ZmDof1*. Two nitrogen treatments were applied: a moderate nitrogen: 95.3 kg/ha residual + applied N, and a low nitrogen: 53.8 kg/ha residual. The experiment was arranged as a split plot design with nitrogen treatment as a whole plot and four independent events and a wild type as split plots. A total of Six blocks were used in this design, three for each nitrogen treatment. A total of 600 seeds were planted per plot distributed in six rows. Each plot was 3.0 × 3.0 m. *ZmDof1* expression, PEPC enzymatic activity and chlorophyll were measured at anthesis as previously described. Photosynthesis rate, determined with LICOR LI-6400XT Portable Photosynthesis System was also measured at anthesis. Light was supplied by 6400-02 LED lights at 1500 μmol m^−2^ s^−1^, 55 ± 5 relative humidity and 400 ppm of CO_2_. Initial measurements of fresh and dry biomass were determined by harvesting 0.3 m of row space per plot. The above ground tissue was collected and dried at 100°C during 2 days. Height was determined at physiological maturity and tissue from one meter per row was collected to determine fresh and dry biomass. Yield per plot and 100 seed weight were determined at harvest.

### Plant material, RNA isolation, and affymetrix microarray hybridization

The wheat independent event pPTN1034 NN569-3-5-1 T_2_ (UD-2) carrying the *ZmDof1* cassette driven by the constitutive promoter UBI4 was selected for microarray analysis. Transgenic progeny and wild type (CBO37) were grown side by side under greenhouse conditions (10/14 h photoperiod and day/night temperatures of 29°/24°C). Initial identification of transformed plants was done by PCR. Genomic DNA was isolated as previously mentioned. Amplification of the synthetic *ZmDof1* from pPTN1034 events was done with the primer set 5′GCTGGGAGGTGTTGTAGTTGTT-3′ and 5′ TTCGCTTACTCTTCTGGTCCTC-3′. The PCR reaction contained ~100 ng DNA, 0.4 μM of each primer, and 1X GoTaq® Green Master Mix. The conditions used for PCR were 95°C for 2 min, 35 cycles of 95°C for 20 s, 52°C for 30 s and 72°C for 1 min, and a final extension of 72°C for 10 min. PCR products were separated by electrophoresis on a 0.8% agarose gel. Tissue from flag leaves was collected to confirm *ZmDof1* expression by northern blot and enzymatic activity of PEPC were determined from selected individuals used for microarray analysis. A total of four samples were obtained for microarray analysis (two transgenic and two wild type). Each sample consisted of pooled RNA from flag leaves from three different plants. RNA was isolated using TRIzol® reagent (Invitrogen cat. # 10296-028) as previously described, and treated with RNase Free DNase Qiagen (Cat #79254). RNA was cleaned using Qiagen RNeasy Mini Kit (P/N 74104). The University of Nebraska's Genomics Core Research Facility carried out the Affymetrix wheat genome microarray hybridizations. Antisense cRNA was obtained from double stranded cDNA synthetized using Affymetrix on-cycle cDNA synthesis kit (Cat #900493). Labeled samples where hybridized to Affymetrix GeneChip® Wheat Genome Arrays (cat # 900558), and stained with streptavidin-phycoerythrin in an Affymetrix GeneChip Fluidics Station 450. Gene chip image acquisition and data processing was obtained using Affymetrix GCS 3000 7G scanner.

### Analysis of microarray data

Transcript profiling analysis was conducted using Affymetrix GeneChip® Wheat Genome Arrays (cat # 900558). The Affymetrix wheat genome array is composed by 61,127 probe sets that represent 55,052 transcripts distributed in all 42 chromosomes. Using robust multiarray average (RMA) method, each set of raw data was background corrected, log_2_-transformed and normalized through the *Affy* package from Bioconductor (Gautier et al., 2004). The *limma* package was utilized to conduct empirical Bayes moderated *t*-test (Ritchie et al., 2015). *P*-values were adjusted to control the false discovery rate (FDR) using Storey and Tibshirani method (Storey and Tibshirani, 2003). Annotations from differentially expressed transcripts were blasted with the rice, *Arabidopsis* and *Brachypodium* genome. The microarray data was deposited in NCBI's Gene Expression Omnibus (Edgar et al., 2002) under the GEO series accession number GSE84330.

### Quantitative real time PCR (qRT-PCR) analyses

Six up-regulated and four down-regulated genes were selected to validate the microarray data by qRT-PCR. Primers were designed using the tool primer3 based on the sequence of each differentially expressed probe accessible on the Affymetrix website (Table S3). RNA was isolated as previously described, treated with RNase Free DNase Qiagen (Cat #79254) and cleaned with Qiagen RNeasy Mini Kit (P/N 74104). cDNA was synthesized from 1 μg RNA using SuperScriptII Platinum Two-Step qRT-PCR kit (Invitrogen cat # 11735). Quantitative real time PCR (qRT-PCR) was performed in a BioRad iCycler using SYBRGreenER™ qPCR SuperMix (Invitrogen cat# 11761) following manufacturer's instructions. Wheat actin gene was used as internal reference to normalize Ct-values obtained for each gene. qRT-PCR data was analyzed based on Dussault and Pouliot (2006) method.

### Statistical analysis

All data from nitrogen evaluations were analyzed using analysis of variance (ANOVA) procedures of SAS® 9.3 software (SAS Institute Inc., Cary, NC 27513-2414 USA). Hydroponics and field experiments were evaluated as split-plot experimental designs with two nitrogen levels as the main plots and seven genotypes as subplots respectively. The soil-less potting phenotyping systems for wheat and sorghum were evaluated as randomized complete block designs with two levels of nitrogen and seven genotypes, respectively. Statistical analysis for PEPC activity and qRT-PCR was determined with *t*-tests using GraphPad Prism 6 software (La Jolla, CA 92037 USA).

## Results

### Generation and molecular characterization of transgenic events

Two expression cassettes were assembled with a synthetic version of the *ZmDof1*. The first harbored the sugarcane polyubiquitin promoter, UBI4, and the second carried the maize rubisco subunit 1 promoter (*rbc*S1). The final binary vectors are designated pPTN1034 and pPTN1037, for the UBI4 and *rbc*S1 cassettes, respectively (Figure 1A). An *Agrobacterium*-mediated gene transfer protocol was used for both wheat (Clemente and Mitra, 2004) and sorghum (Howe et al., 2006) transformations. A total of 13 wheat events and 21 sorghum events were generated from pPTN1034, and 8 wheat events and 13 sorghum events were produced from pPTN1037 transformations. *ZmDof1* transcript accumulation on selected wheat events is shown in Figure 1B. Among the transgenic wheat events derived, a subset, was selected for further characterizations. These are designated, pPTN1034-NN569-2-2-1, pPTN1034-NN569-3-5-1, and pPTN1034-NN569-1-2-1 henceforth referred to as UD-1, UD-2, and UD-3, respectively. While the selected *rbc*S1/Dof1 transgenic wheat events are pPTN1037-NN573-1-11-1, pPTN1037-NN581-2-4-2, and pPTN1037-NN581-2-1-1 abbreviated as RD-1, RD-2, and RD-3, respectively. Transgenic expression of *ZmDof1* induces the up-regulation and subsequent elevation in endogenous activity of phospho*enol*pyruvate carboxylase (PEPC) in rice and Arabidopsis (Yanagisawa et al., 2004; Kurai et al., 2011). Results from this study also showed a significant increase in PEPC activity in wheat (Figure 1C) in the transgenic events carrying either the constitutive or tissue specific regulatory elements. However, transcript accumulation did not correlate with PEPC activity (Figures 1B,C). Among the transgenic sorghum events a subset was selected for further characterizations. Three events bearing the constitutive expression of *ZmDof1* designated as pPTN1034 ZG160-1-1b, pPTN1034 ZG161-1-1a, and pPTN1034 ZG176-3-9a referred to as S-UD-1, S-UD-2, and S-UD-3, respectively. Additionally, the selected *rbc*S1/Dof1 transgenic sorghum events are pPTN1037 ZG164-1-4a, pPTN1037 ZG164-1-7a, and pPTN1037 ZG164-3-7a designated as S-RD-1, S-RD-2, and S-RD-3, respectively. The *ZmDof1* transcript accumulation is observed in the transgenic sorghum events (Figure 1D). However, the up-regulation of PEPC by *ZmDof1* was not evident as the enzymatic activity of PEPC remained unaltered in relation to the control plants, S-WT, (Figure 1E).

### Impact of *ZmDof1* expression in wheat on NUE

The selected *ZmDof1* wheat events were evaluated in three 5-week hydroponics studies under full nitrogen (15 mM N) or low nitrogen conditions (0.3 mM N). The data revealed alterations in biomass accumulation, in the events grown under sufficient N conditions, was impacted by promoter regulating *ZmDof1*. Constitutive *ZmDof1* triggered detrimental effects including distinct reduction in both root and shoot dry biomass (Table 1). The total dry biomass was reduced up to 47% when compared to control plants. In contrast, dry biomass accumulation of two *rbc*S1/Dof1 events was elevated by 23 and 29% (shoots/roots, respectively; Table 1). Under low nitrogen conditions, the biomass of the transgenic events did not differ from the WT. However, a similar tendency regarding a stunted phenotype in the constitutive *ZmDof1* events was observed along with decreases in NUE (Dry biomass per N supplied). In contrast, tissue specific expression of *ZmDof1* under the control of *rbcS1* led to increases of NUE under low nitrogen (Table 1). Constitutive *ZmDof1* also induced reduction in plant height (Table 2). A common phenotype observed in the transgenic events was a significant increase in tillering. This phenotype together with height was observed under both N regimes (Table 2). Shoot total nitrogen content, and chlorophyll levels varied across events/treatment, but no correlation with the respective transgenic alleles (Table 2).

**Table 1:** **Biomass analysis of *ZmDof1* wheat events**.

**N trt**	**Line**	**Shoot DW**		**Root DW**		**Total DW**		**NUE**	
		**gr**	**% WT**	**gr**	**% WT**	**gr**	**% WT**	**Index**	**% WT**
High N	WT	3.4 ± 0.5^b^		0.8 ± 0.2^b^		4.3 ± 0.6^b^		0.28 ± 0.04^a^	
	UD-1	2.4 ± 0.4^c^	69	0.5 ± 0.1^bcd^	64	2.9 ± 0.5^c^	68	0.19 ± 0.04^a^	68
	UD-2	2.3 ± 0.5^c^	65	0.5 ± 0.1^cd^	61	2.7 ± 0.6^c^	65	0.18 ± 0.04^a^	65
	UD-3	1.8 ± 0.2^c^	54	0.4 ± 0.1^d^	49	2.2 ± 0.3^c^	53	0.15 ± 0.02^a^	53
	RD-1	3.0 ± 0.4^b^	88	0.8 ± 0.2^bc^	100	3.8 ± 0.6^b^	90	0.26 ± 0.04^a^	90
	RD-2	4.1 ± 0.5^a^	119	1.1 ± 0.2^a^	141	5.2 ± 0.7^a^	123	0.35 ± 0.05^a^	123
	RD-3	4.3 ± 0.6^a^	124	1.2 ± 0.2^a^	152	5.5 ± 0.8^a^	129	0.37 ± 0.05^a^	129
Low N	WT	1.8 ± 0.2^ab^		0.6 ± 0.1^b^		2.3 ± 0.3^abc^		7.8 ± 1.11^b^	
	UD-1	1.6 ± 0.2^ab^	91	0.5 ± 0.1^bcd^	88	2.1 ± 0.3^bc^	90	7.0 ± 0.86^bc^	90
	UD-2	1.4 ± 0.3^b^	81	0.5 ± 0.1^cd^	85	1.9 ± 0.4^c^	82	6.4 ± 1.44^bc^	82
	UD-3	1.3 ± 0.2^b^	73	0.4 ± 0.1^d^	62	1.6 ± 0.2^c^	70	5.5 ± 0.69^d^	70
	RD-1	1.6 ± 0.2^ab^	91	0.6 ± 0.1^bc^	95	2.2 ± 0.4^bc^	92	7.2 ± 1.22^b^	92
	RD-2	2.3 ± 0.3^a^	128	0.8 ± 0.1^a^	137	3.1 ± 0.4^a^	130	10.2 ± 1.44^a^	130
	RD-3	2.2 ± 0.2^a^	123	0.7 ± 0.1^a^	123	2.9 ± 0.3^ab^	123	9.6 ± 0.88^a^	123

*Shoot dry weight, root dry weight, total dry weight, and nitrogen use efficiency (NUE, Dry biomass per N supplied) of control plants (WT). gr refers to grams; three UBI4/Dof1 transgenic events (UD-1, UD-2, UD-3); and three rbcS1/Dof1 transgenic events (RD-1, RD-2, RD-3). Plants were grown hydroponically for 5 weeks under 15 mM N (High N) or 0.3 mM N (Low N). Data expressed as mean ± standard error from three independent experiments. Different letters indicate statistical differences (p < 0.05) within N trt*.

**Table 2:** **Physiological components of *ZmDof1* wheat events**.

**N trt**	**Line**	**Height**		**Tillers**		**Shoot total N**		**Chlorophyll**	
		**cm**	**% WT**	**Number**	**% WT**	**%**	**WT%**	**μmol m^−2^**	**% WT**
High N	WT	53 ± 3.0^ab^		3.9 ± 0.4^b^		3.4 ± 0.1^a^		651 ± 51^cb^	
	UD-1	48 ± 2.6^bc^	90	4.4 ± 0.7^a^	114	3.4 ± 0.3^ab^	99	655 ± 27^b^	101
	UD-2	44 ± 3.9^cd^	83	4.8 ± 0.7^a^	123	3.3 ± 0.1^abc^	96	605 ± 82^c^	93
	UD-3	43 ± 1.3^d^	81	4.3 ± 0.6^a^	111	3.2 ± 0.1^c^	93	750 ± 32^a^	115
	RD-1	54 ± 3.0^a^	101	4.7 ± 0.5^a^	120	3.5 ± 0.2^a^	100	620 ± 19^bc^	95
	RD-2	55 ± 3.0^a^	103	4.9 ± 0.7^a^	126	3.2 ± 0.1^bc^	94	648 ± 26^bc^	100
	RD-3	54 ± 2.0^a^	102	4.8 ± 0.6^a^	123	3.3 ± 0.1^abc^	96	676 ± 24^b^	104
Low N	WT	56 ± 3.1^ab^		1.7 ± 0.2^b^		2.0 ± 0.2^b^		578 ± 14^cb^	
	UD-1	52 ± 2.7^bc^	94	2.7 ± 0.2^a^	160	2.2 ± 0.3^ab^	109	583 ± 13^b^	101
	UD-2	47 ± 3.6^cd^	84	2.6 ± 0.3^a^	153	2.0 ± 0.2^b^	99	506 ± 39^c^	87
	UD-3	44 ± 2.9^d^	80	2.4 ± 0.3^a^	147	2.3 ± 0.3^a^	118	640 ± 16^a^	111
	RD-1	61 ± 2.7^a^	110	2.2 ± 0.2^a^	133	2.0 ± 0.2^b^	101	534 ± 12^bc^	92
	RD-2	60 ± 2.3^a^	107	2.6 ± 0.2^a^	153	2.0 ± 0.2^b^	100	534 ± 14^bc^	92
	RD-3	63 ± 2.2^a^	113	2.9 ± 0.3^a^	173	2.0 ± 0.2^b^	99	568 ± 6^b^	98

*Chlorophyll, height, tillers, and shoot total nitrogen of control plants (WT); three UBI4/Dof1 transgenic events (UD-1, UD-2, UD-3); and three rbcS1/Dof1 transgenic events (RD-1, RD-2, RD-3). Plants were grown hydroponically for 5 weeks under: 15 mM N (High N) or 0.3 mM N (Low N). Data expressed as mean ± standard error from three independent experiments. Different letters indicate statistical differences (p < 0.05) within N trt*.

### Field evaluations of *ZmDof1* wheat events

A field trial was conducted during spring-summer of 2015 in Mead, Nebraska on the selected wheat events. The transgenic events and WT were evaluated in a split plot design, with nitrogen factor as a whole plot and transgenic event and WT as a split plot. Plants were randomized in either three blocks for moderate nitrogen: 94.1 kg/ha residual + applied N, or three blocks for low nitrogen: 53.2 kg/ha residual. Due to sufficient soil N levels, the analysis of variance conducted for different physiological components indicated no N effect across genotype, the data is presented as six blocks per transgenic event and WT without considering the nitrogen effect.

**Figure 2:**
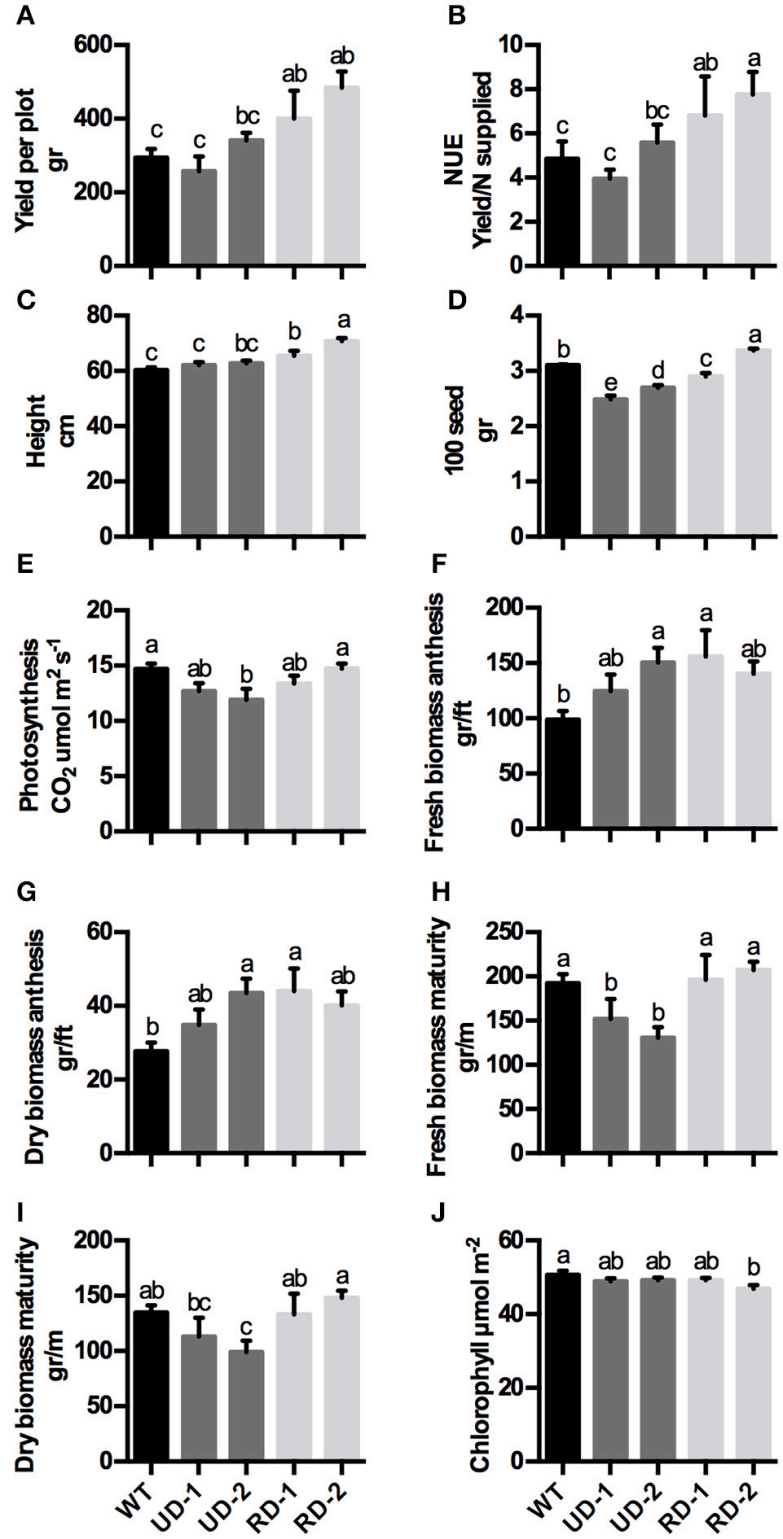
**Physiological components of wheat plants expressing *ZmDof1* under field conditions. (A)** Yield per plot, **(B)** NUE index (Yield/N supplied), **(C)** Height, **(D)** 100 seed weight, **(E)** photosynthesis rate, **(F)** Fresh biomass at anthesis, **(G)** Dry biomass at anthesis, **(H)** Fresh biomass at maturity. **(I)** Dry biomass at maturity, and **(J)** Chlorophyll concentration of control plants (WT)-black bars; two UBI4/Dof1 transgenic events (UD-1, UD-2)-gray bars, and two *rbc*S1/Dof1 transgenic events (RD-1, RD-2)-silver bars. Plants were grown in the field under moderate nitrogen: 95.3 kg/ha residual + applied N, and a low nitrogen: 53.8 kg/ha residual (*n* = 3 plots per treatment combination). Analysis of variance indicated no significant differences on nitrogen treatment; therefore the data is expressed as mean ± *SE* of 6 plots. Different letters indicate statistical difference (*p* < 0.05).

The results from this field study indicated no changes in yield per plot with constitutive events, however, the tissue specific *ZmDof1* events displayed a 36 and 64% increase relative to WT plots (Figure 2A). Moreover, increases in both NUE index and height parameters were observed in the RD *ZmDof1* events (Figures 2B,C). Despite the changes in biomass accumulation during vegetative growth (Figures 2F,G), the leaf net photosynthetic rate was reduced in the constitutive events, which translated to a reduced biomass at maturity and reduced 100 seed weight (Figures 2D–I). However, these agronomic and physiological changes did not affect yield, suggesting that the constitutive events generate more seeds per plot than the WT. The RD wheat events exhibited an increase in biomass between 45 and 59% relative WT, during early vegetative stages, but at maturity biomass was similar to the WT (Figures 2F–I). No change in chlorophyll content was observed between transgenic events and WT lines (Figure 2J).

### Promoter regulating *ZmDof1* induces contrasting phenotypes in both wheat and sorghum

**Figure 3:**
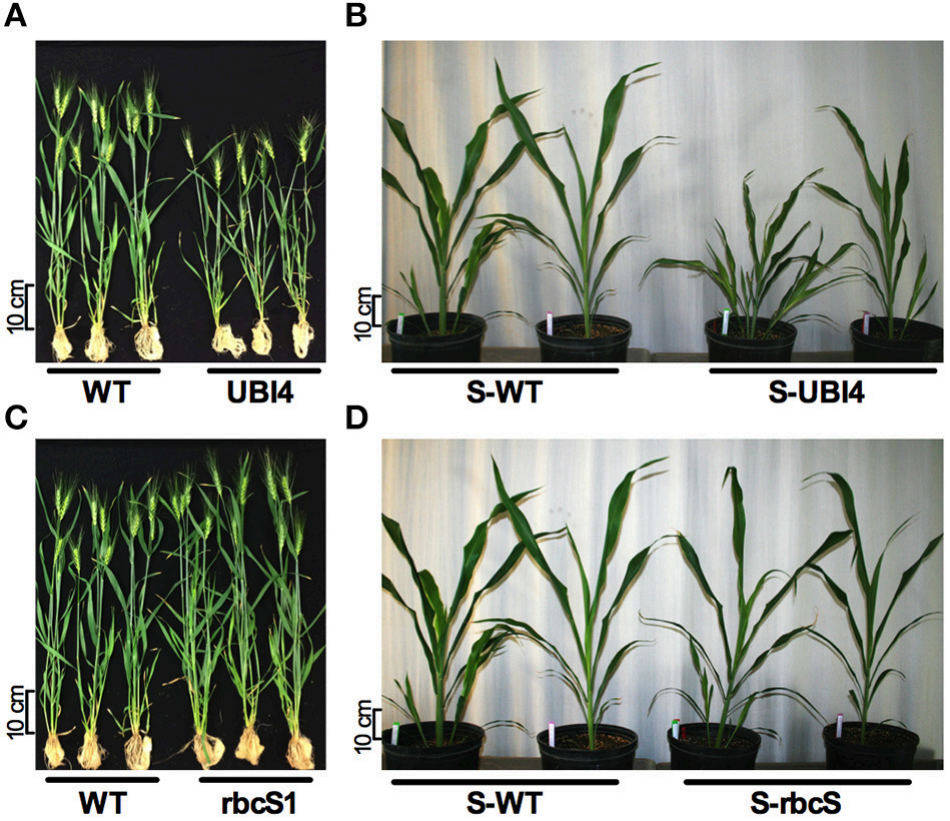
**Comparison of the promoter effect on *ZmDof1* expression in wheat and sorghum plants. (A)** Effect of the UBI4 promoter in wheat UBI4/Dof1 transgenic events (UBI4) compared to control plants (WT). **(B)** Effect of the UBI4 promoter in sorghum UBI4/Dof1 transgenic events (S-UBI4) compared to control plants (S-WT). **(C)** Effect of the *rbc*S1 promoter in wheat *rbc*S1/Dof1 transgenic events (rbcS1) compared to control plants (WT). **(D)** Effect of the *rbc*S1 promoter in sorghum *rbc*S1/Dof1 transgenic events (S-rbcS1) compared to control plants (S-WT). Wheat plants were grown hydroponically for 5 weeks. Sorghum plants were grown in pots for 6 weeks.

**Figure 4:**
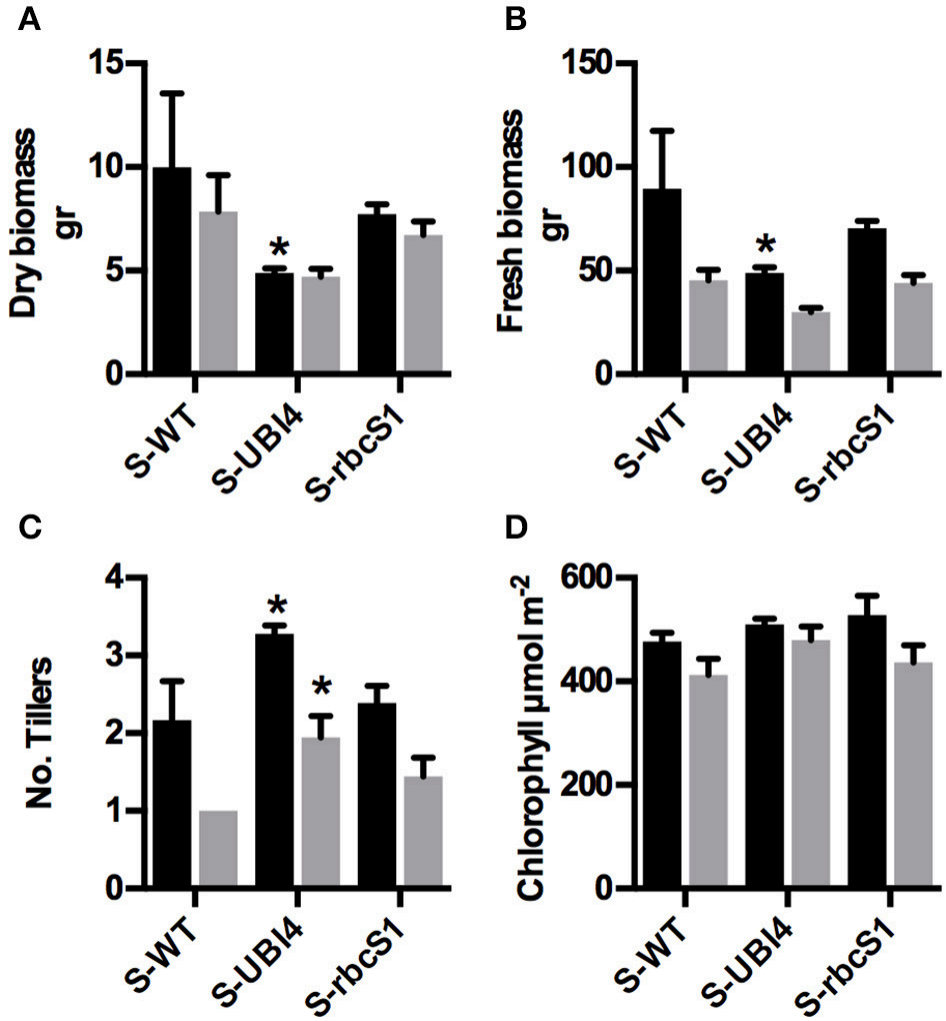
**Comparison of the promoter effect on *ZmDof1* expression in physiological components of sorghum plants. (A)** Dry biomass above ground, **(B)** Fresh biomass above ground, **(C)** Number of tillers, and **(D)** Chlorophyll of control plants (S-WT); pooled data of three UBI4/Dof1 transgenic lines (S-UBI4); and pooled data of three *rbc*S1/Dof1 transgenic lines (S-rbcS1). Plants were grown in pots for 6 weeks under 15 mM N (Black bars) or 0.75 mM N (Silver bars). Data expressed as mean ± *SE* of two independent experiments. Each experiment had three plants per nitrogen treatment and line combination. Asterisks indicate significant differences from the control (*p* < 0.05).

Datasets obtained from hydroponic and field experiments with the *ZmDof1* wheat events revealed differential phenotypic outcomes between to the two expression cassettes. For example, under controlled hydroponics conditions the UD events exhibited a stunted and abnormal phenotype, while RD events displayed positive response in parameters monitored (Table 1, Figure 2). While under field environment a negative impact on biomass at maturity, NUE, and 100 seed weight was observed in the UD events. To gain insight on how the phenotypic outcomes triggered by *ZmDof1* expression in a C_3_ monocot would translate to a C_4_ monocot, three UD and RD sorghum events were selected for a 6 week greenhouse study sown in 40/40/20 vermiculite/sand/MetroMix 200 matrix, under two N treatments (15 mM N and 0.75 MM N). Data were pooled by *ZmDof1* cassette to highlight the effect of the promoter under the two N regimes. The data revealed no changes in PEPC enzymatic activity relative to WT (Figures 1D,E). However, the constitutive *ZmDof1* sorghum events displayed enhanced tiller production, stunting, and reduced biomass accumulation (Figures 3A,B, 4A–C). No changes in chlorophyll content, tiller number and biomass parameters were observed in the RD sorghum events, relative to WT, under the conditions of this study (Figures 3D, 4A–D).

### Transcript profiling changes in UD *ZmDof1* wheat

**Figure 5:**
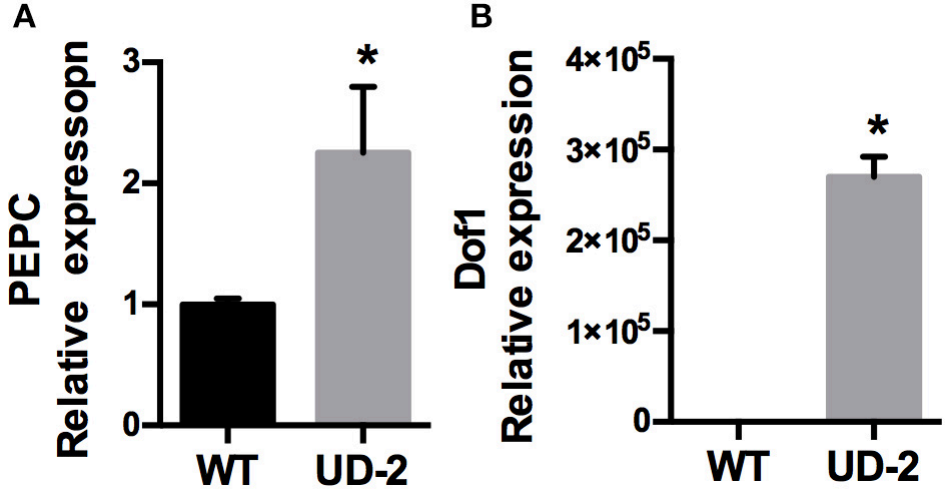
**Relative transcript abundance of PEPC and Dof1**. Expression analysis using qRT-PCR of **(A)** PEPC (GenBank: AJ007705.1) and **(B)** Dof1 of the UBI4/Dof1 transgenic event UD-2 compared to wheat control plants (WT). Data expressed as mean ± SE (*n* = 2); each sample (*n*) was leaf tissue pooled from three different wheat plants at anthesis. Asterisks indicate significant differences from the control (*p* < 0.05).

**Figure 6:**
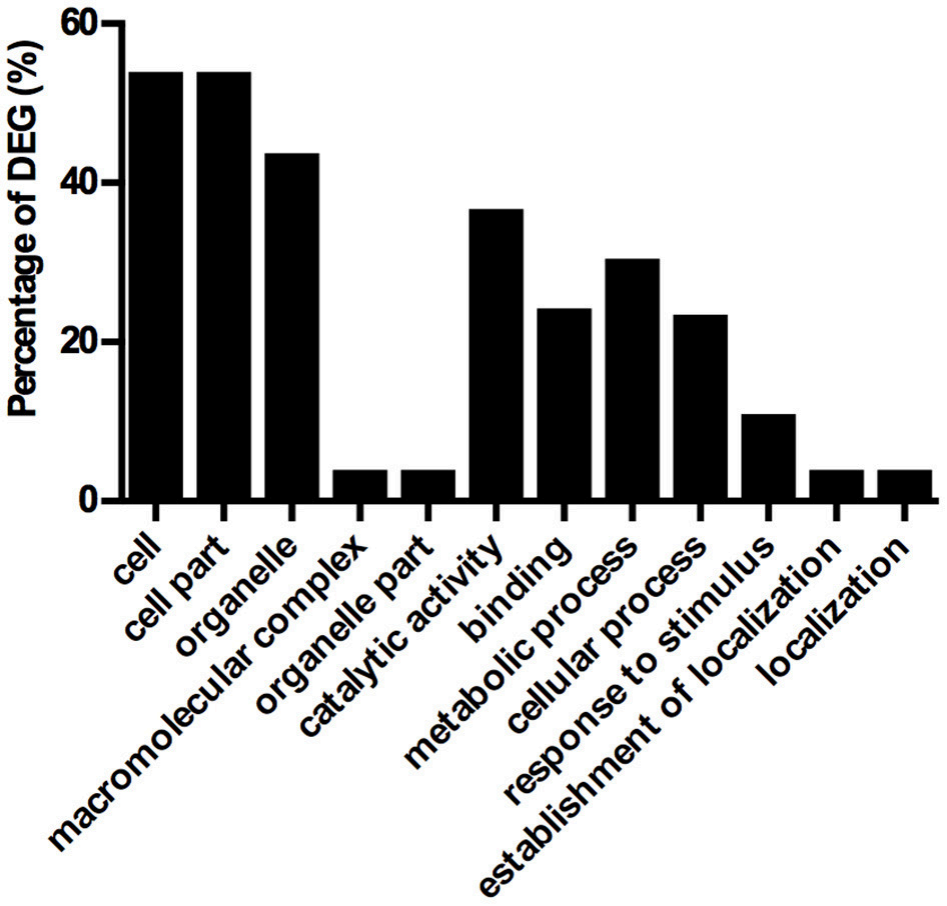
**GO annotation of differentially expressed genes**. Gene Ontology functional enrichment analysis of differentially expressed genes using AgriGO web-based tool. Percentage of differentially expressed genes (DEG) exhibiting transcriptional changes in three main categories: cellular component, molecular function, and biological process.

**Figure 7:**
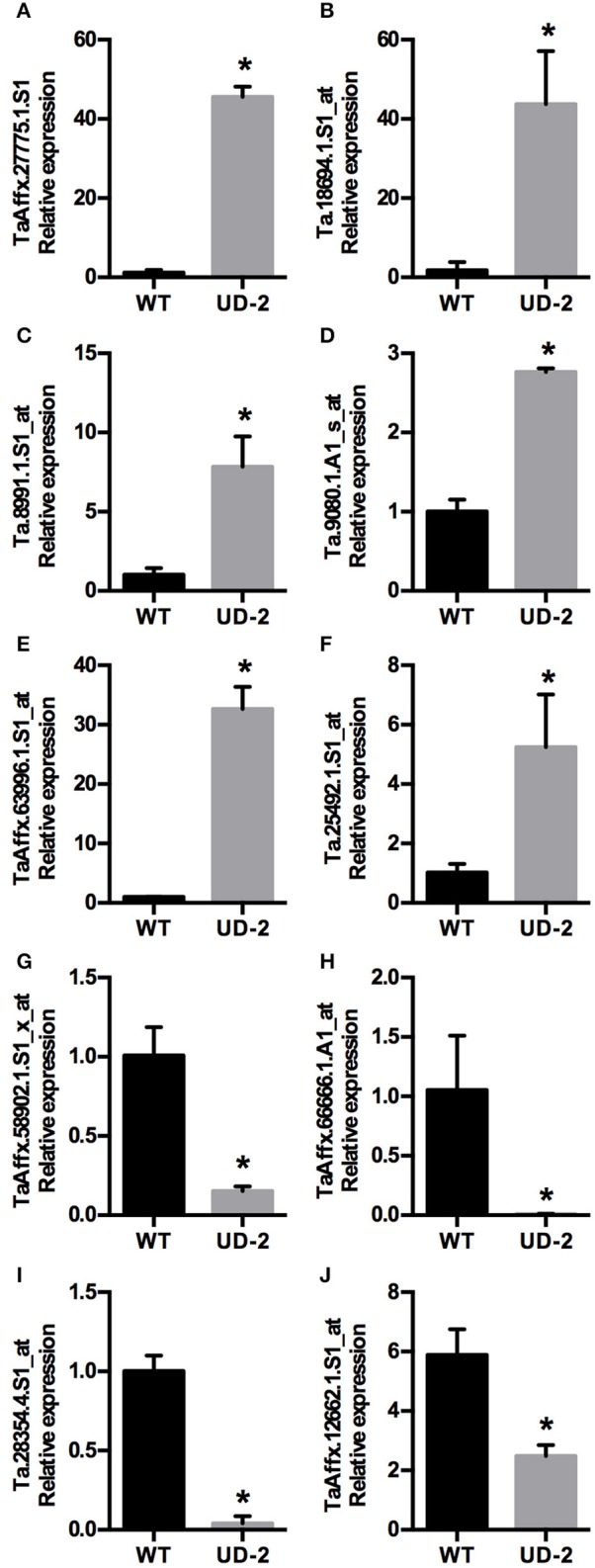
**Validation of microarray data**. Expression analysis using qRT-PCR of **(A–F)** Six up regulated, and **(G–J)** Four down regulated genes of the UBI4/Dof1 transgenic event UD-2 compared to wheat control plants (WT). Data expressed as mean ± *SE* (*n* = 2); each sample (*n*) was leaf tissue pooled from three different wheat plants at anthesis. Asterisks indicate significant differences from the control (*p* < 0.05).

Differentially expressed genes (DEG) were monitored in the constitutive *ZmDof1* event, UD-2, via microarray analysis (2 UD-2 and 2 WT). RNA pools from flag leaves across three different plants, WT and UD-2, were prepared. Expression of a PEPC and the transgene, *ZmDof1*, via qRT-PCR, was carried out on the individuals utilized in the microarray. The outcome of these specific expression profiles is shown in Figure 5. Transcript profiling analysis was conducted using Affymetrix GeneChip® Wheat Genome Arrays (cat # 900558). A differentially expressed transcript is defined as having a mean signal intensity ratio (log_2_) significantly different to the WT at an adjusted *p*-value (< 0.05). Based on this criterion 137 up-regulated and 94 down-regulated transcripts were identified. Given the wheat genome is not completely annotated, the DEGs were blasted against the rice, and Arabidopsis genomes. Results from 50 up-regulated and 50 down-regulated transcripts are shown in Tables S1, S2. Gene Ontology (GO) analysis was conducted to evaluate potential functions of these DEGs using the agriGO online service (Du et al., 2010). Assignment for each differentially expressed transcript to a category based on functional enrichment analysis was partitioned to three main categories: cellular component, molecular function, and biological processes (Figure 6). The DEGs assigned to cellular component partition were sub-classified into five functional categories, with the majority falling under three sub-classes, cell, cell part (54% each), and organelle (44%). Within the molecular function partition, the DEGs were sub-classified into catalytic activity (37%) and binding (24%). This analysis also indicated five sub-categories under biological processes including metabolic processes (30%), cellular processes (23%), and response to stimulus (11%). Approximately, 25% of the DEGs were unknown and unclassified proteins. Annotations from 50 upregulated and 50 downregulated transcripts are shown in Tables S1, S2, respectively. Overall, only three DEGs involved in carbon and nitrogen metabolism were significantly modulated: an aspartate amino transferase (Ta.5314.2.S1_a_at, similar to AT2G30970.2 from *Arabidopsis*), which was slightly up-regulated, a down-regulated glycosyl hydrolase and a putative PEPC (Table S2). As a means to validate the microarray data, nine differentially expressed transcripts were randomly selected and subsequently quantified via qRT-PCR (Figure 7). Six up-regulated and four down-regulated transcripts exhibited similar differential expression as observed in the microarray analysis reflecting a positive reliability of the microarray dataset (Figure 7).

## Discussion

A transcription factor strategy was implemented to gain insight on the effects of the modulation of carbon skeleton production and nitrogen use efficiency (NUE) in wheat and sorghum. The ZmDof1 transcription factor has been shown to enhance growth through global activation of carbon skeleton metabolism, a hallmark of which is the enhancement of PEPC activity (Yanagisawa et al., 2004; Kurai et al., 2011). In C_4_ plants, PEPC is compartmentalized within mesophyll cells, and is regulated both transcriptionally and post-transcriptionally (Chollet et al., 1996), with a direct link to photosynthesis, where it plays a pivotal role in down-stream metabolism ensuing carbon capture. In C_3_ plants, on the other hand, PEPC activity is more directly linked with N/C networks, where it provides carbon substrates to the TCA cycle (Chollet et al., 1996; Jeanneau et al., 2002; O'Leary et al., 2011).

Expression of *ZmDof1* in the C_3_ crop wheat modulated transcript accumulation of PEPC and enhanced its enzymatic activity showing consistency with the results in *Arabidopsis* and rice. However, PEPC activity elevation did not appear to be correlated directly with *ZmDof1* transcript level *per se*, for both *ZmDof1* cassettes triggered similar boost in PEPC activity, yet wheat events carrying the constitutive cassette displayed higher transcript accumulation (Figure 1). Moreover, this elevation in PEPC activity was not observed in the C_4_ sorghum events, in which high levels of *ZmDof1* transcript were detected (Figure 1), under the assay conditions utilized in this study.

A common phenotype that was observed in both wheat and sorghum events expressing the constitutive *ZmDof1* cassette (pPTN1034), was a reduction in biomass, along with degree of stunting (Figure 3), which a similar outcome was observed in rice (Kurai et al., 2011). These off-type outcomes in rice harboring a constitutive *ZmDof1* allele were hypothesized to be associated with elevation in PEPC activity. The data monitoring PEPC activity in constitutive wheat and sorghum events is not suggestive of such a relationship between PEPC and stunting (Figures 1, 3). Transcript profiling of the constitutive *ZmDof1* event, UD2, which displays a stunting phenotype, revealed significant modulation in gene calls associated with glutaredoxin function (Table S1), changes in expression of which will likely impact cellular redox homeostasis (Ziemann et al., 2008; Meyer et al., 2012). To this end, ectopic expression of the rice glutaredoxin *OsGRX6* led to a number of off type phenotypes including stunting, and reduction in above and below ground vegetative biomass (El-Kereamy et al., 2015). These developmental changes in rice were associated with alterations in N metabolism and growth regulator status of tissues (El-Kereamy et al., 2015). Moreover, direct elevation of PEPC activity in wheat through introduction of a maize PEPC allele revealed no phenotypic off types associated with growth or architecture (Hu et al., 2012). Clearly, the *ZmDof1* TF can trigger a diverse array of metabolic and potentially architectural changes. In regards to the latter, a gene call significantly up-regulated in the UD2 event is a microtubule associated protein (MAP; Table S1; Figure 7D), which the MAP family is intimately involved in depositions of cell walls and vascular tissue patterning (Pesquet et al., 2011). Hence, while *ZmDof1* may directly impact central C metabolism *in planta* through modulation of PEPC activity, the direct link with growth defects attributed to boost in enzymatic activity is tenuous.

The second off type observed UD2 wheat event is significant reduction in photosynthetic capacity under field conditions (Figure 2E). This phenotype may be partially explained by the reduction in expression of gene calls for photosystem II subunits (Table S2). A confounding data point from the microarray analyses on this event is the observed significant down-regulation of a gene call for PEPC (Table S2), which was not in agreement with the enzymology (Figure 1C). The qRT-PCR confirmation assay for this chip ID, TaAffx.12662.1 (Table S1), which possesses high identity to both *Brachypodium distachyon* (Genbank acc. Xm_003565281.3) and rice (*Oryza sativa*; Genbank acc. Xm_015793803.1) PEPC genes, was in agreement with the microarray analysis (Figure 7J). However, a primer set designed toward the wheat PEPC (Genbank acc. AJ007705.1) when utilized in a qRT-PCR assay conducted on RNA samples from UD2 revealed a boost in expression (Figure 5A) in accordance with the enzymology results.

Detrimental effects in plants with a high expression of foreign genes in all tissues has been documented (Cheon et al., 2004). In wheat, the UBI4 promoter drives a strong constitutive gene expression of *ZmDof1* leading to numerous detrimental effects. In contrast, the rbcS promoter from maize, which drives a light-regulated gene expression specifically to the mesophyll cells in the leaf blades, and leaf sheaths in C3 crops (Matsuoka et al., 1994), resulted in positive agronomic effects in wheat. The off type characteristics observed in the constitutive wheat events were differentially manifested across treatments/environment. For example, under controlled hydroponics conditions, sufficient N treatment, the constitutive events displayed stunting, with reduced biomass accumulation relative to controls, while those under low N treatment had similar biomass (Tables 1, 2). However, the same events under field conditions did not display stunting, but biomass at maturity was impacted, with suppression in photosynthesis (Figure 2). Noteworthy, lineages of the constitutive events consistently segregated in a 1:1 ratio, suggestive of a non-transmission of the transgenic allele through one of the gametes, which may explain the inability to recover homozygous lineages in constitutive *ZmDof1* rice events (Kurai et al., 2011).

Homozygous lineages derived from the RD events (pPTN1037), harboring the light regulated, tissue specific promoter element (Nomura et al., 2000; Sattarzadeh et al., 2010) were identified, and off type characteristics mitigated (Table 1), while maintaining the tiller number increases similar to that observed in the constitutive events (Table 2). Importantly agronomic attributes including enhancement in NUE, 100 seed weight, estimated plot yield were realized by utilizing the *rbc*S1 promoter to govern *ZmDof1* expression in wheat (Figure 2).

NUE is a complex quantitative trait, which is influenced by environmental factors that affect all components of the process, from N uptake and assimilation to remobilization (Han et al., 2015). In both sorghum and wheat genetic gains in yield have come at no expense to grain protein quality or quantity, reflecting a concomitant stepwise improvement in NUE realized by the ongoing respective breeding efforts for these two important grain crops (Cormier et al., 2013; Ciampitti and Prasad, 2016). The tools of biotechnology serve as a resource for plant breeders to draw upon for the introduction of novel genetic alleles in the germplasm pool that compose their respective programs. NUE remains a high priority physiological phenotype in agriculture. The rationale for NUE importance, is underpinned by cost of goods of petro-based fertilizers, and the negatives linked to N leaching into watersheds that ultimately has regional connotations, all of which negatively affect the three prongs of sustainability, profit, environment, and society.

As a means to address NUE through biotechnology several approaches have been explored including modification of root architecture, enhancing nitrate, and ammonium transport and by manipulating key genes regulating the balance of nitrogen and carbon metabolism (Liu et al., 1999; Fuentes et al., 2001; Oliveira et al., 2002; Good et al., 2004, 2007; Martin et al., 2006; Giannino et al., 2007; Shrawat et al., 2008; Brauer et al., 2011; McAllister et al., 2012; Xu et al., 2012; Seger et al., 2015). Most of these studies involve single gene strategies to address this complex trait. The outcomes of these approaches have translated to modest changes in N metabolism and growth. However, many led to either no observable changes or detrimental phenotypic effects attributed to imbalances in important metabolic pathways that are governed by multiple interacting N/C metabolic fluxes. TF strategies designed to address NUE have also been explored. For example, ectopic expression of the nitrate-inducible NAC transcription factor TaNAC2-5A in wheat enhanced root growth, nitrate influx rate and increased wheat yield (He et al., 2015). Introduction of a transgenic allele of the endogenous NAC transcription factor NAM-B1 has been shown to accelerate senescence, increase nutrient remobilization from source to sink organs that translated to improved outputs for protein, Zn and Fe content in wheat (Uauy et al., 2006).

Phenotyping for NUE in crops under controlled environmental conditions has great value in elucidating the mechanistic underpinnings for the trait. However, given the genetic complexity underlying NUE in crops, it is imperative that such sources of novel genetic variation for the trait be phenotype under field conditions. The efforts communicated herein translate the *ZmDof1* strategy, as a means to alter N/C networks to affect NUE (Yanagisawa et al., 2004; Kurai et al., 2011; Lin et al., 2013), to wheat and sorghum. The evaluated *ZmDOF1* cassettes had a common outcome in both cereals, promoter activity impacted phenotype (Figure 3), highlighting the importance vector design of transgenic alleles. The use of a light controlled promoter element, mitigated the observed off types, in both crops, induced by constitutive *ZmDof1* expression. The small-scale field trial conducted with the selected RD wheat events displayed significant boost in wheat grain harvest (Figure 2), with no significant reduction in grain protein (Figure S1). Given the non-mendelian segregation ratios over generations observed in the constitutive UD events, it is challenging to gauge the full agronomic penalty on a per plot basis. Nonetheless, taken together, the information communicated suggest that the *ZmDof1* has the capacity to improve growth and biomass accumulation that lead to higher productivity in wheat. However, the agronomic value of this single TF as a route to capture genetic gains in NUE in grain crops may likely be realized when integrated in a more thoughtful synthetic biology design wherein transgene stacks address the entire NUE process from uptake through flux toward sink, in a holistic manner, with selected endogenous alleles.

## Author contributions

PP, TQ, and TC conducted research, and data analyses. SS, ZG, and NN generated the transgenic events and contributed to molecular analyses. PP, MS, and TEC wrote the article. MS, ID, and TEC supervised project activities.

## Funding

This research was supported by Department of Agronomy and Horticulture, University of Nebraska-Lincoln, Lincoln, NE, 68588. Center for Biotechnology, University of Nebraska-Lincoln, Lincoln, NE, 68588. Center for Plant Science Innovation, University of Nebraska-Lincoln, Lincoln, NE, 68588. Nebraska Wheat Board, Lincoln, NE, 68508.

### Conflict of interest statement

The authors declare that the research was conducted in the absence of any commercial or financial relationships that could be construed as a potential conflict of interest.

## References

[B1] Bao A. Liang Z. Zhao Z. Cai H. (2015). Overexpressing of OsAMT1-3, a high affinity ammonium transporter gene, modifies rice growth and carbon-nitrogen metabolic status. Int. J. Mol. Sci. 16, 9037–9063. 10.3390/ijms16059037 25915023 PMC4463577

[B2] Bradford M. M. (1976). A rapid and sensitive method for the quantitation of microgram quantities of protein utilizing the principle of protein-dye binding. Anal. Biochem. 72, 248–254. 10.1016/0003-2697(76)90527-3 942051

[B3] Brauer E. K. Rochon A. Bi Y. M. Bozzo G. G. Rothstein S. J. Shelp B. J. (2011). Reappraisal of nitrogen use efficiency in rice overexpressing glutamine synthetase1. Physiol. Plant. 141, 361–372. 10.1111/j.1399-3054.2011.01443.x 21214879

[B4] Cai H. Zhou Y. Xiao J. Li X. Zhang Q. Lian X. (2009). Overexpressed glutamine synthetase gene modifies nitrogen metabolism and abiotic stress responses in rice. Plant Cell Rep. 28, 527–537. 10.1007/s00299-008-0665-z 19123004

[B5] Century K. Reuber T. L. Ratcliffe O. J. (2008). Regulating the regulators: the future prospects for transcription-factor-based agricultural biotechnology products. Plant Physiol. 147, 20–29. 10.1104/pp.108.117887 18443103 PMC2330319

[B6] Cheon B. Y. Kim H. J. Oh K. H. Bahn S. C. Ahn J. H. Choi J. W.. (2004). Overexpression of human erythropoietin (EPO) affects plant morphologies: retarded vegetative growth in tobacco and male sterility in tobacco and Arabidopsis. Transgenic Res. 13, 541–549. 10.1007/s11248-004-2737-3 15672835

[B7] Chollet R. Vidal J. O'Leary M. H. (1996). PHOSPHOENOLPYRUVATE CARBOXYLASE: a ubiquitous, highly regulated enzyme in plants. Annu. Rev. Plant Physiol. Plant Mol. Biol. 47, 273–298. 10.1146/annurev.arplant.47.1.273 15012290

[B8] Ciampitti I. A. Prasad P. V. (2016). Historical synthesis-analysis of changes in grain nitrogen dynamics in sorghum. Front. Plant Sci. 7:275. 10.3389/fpls.2016.00275 27014299 PMC4781835

[B9] Clemente T. Mitra A. (2004). Genetic Engineering of Wheat: Protocols and Use to Enhance Stress Tolerance. New York, NY: Food Products Press.

[B10] Cormier F. Faure S. Dubreuil P. Heumez E. Beauchene K. Lafarge S.. (2013). A multi-environmental study of recent breeding progress on nitrogen use efficiency in wheat (*Triticum aestivum* L.). Theor. Appl. Genet. 126, 3035–3048. 10.1007/s00122-013-2191-9 24057081

[B11] Du Z. Zhou X. Ling Y. Zhang Z. Su Z. (2010). agriGO: a GO analysis toolkit for the agricultural community. Nucleic Acids Res. 38, W64–W70. 10.1093/nar/gkq310 20435677 PMC2896167

[B12] Dussault A. A. Pouliot M. (2006). Rapid and simple comparison of messenger RNA levels using real-time PCR. Biol. Proced. Online 8, 1–10. 10.1251/bpo114 16446781 PMC1352391

[B13] Eckert H. La Vallee B. Schweiger B. J. Kinney A. J. Cahoon E. B. Clemente T. (2006). Co-expression of the borage Delta 6 desaturase and the Arabidopsis Delta 15 desaturase results in high accumulation of stearidonic acid in the seeds of transgenic soybean. Planta 224, 1050–1057. 10.1007/s00425-006-0291-3 16718484

[B14] Edgar R. Domrachev M. Lash A. E. (2002). Gene expression omnibus: NCBI gene expression and hybridization array data repository. Nucleic Acids Res. 30, 207–210. 10.1093/nar/30.1.207 11752295 PMC99122

[B15] El-Kereamy A. Bi Y. M. Mahmood K. Ranathunge K. Yaish M. W. Nambara E.. (2015). Overexpression of the CC-type glutaredoxin, OsGRX6 affects hormone and nitrogen status in rice plants. Front. Plant Sci. 6:934. 10.3389/fpls.2015.00934 26579177 PMC4630655

[B16] Food and Agriculture Organization of the United Nations (2009). Declaration of the World Summit on Food Security, WSFS 2009/2 (Rome).

[B17] Fuentes S. I. Allen D. J. Ortiz-Lopez A. Hernandez G. (2001). Over-expression of cytosolic glutamine synthetase increases photosynthesis and growth at low nitrogen concentrations. J. Exp. Bot. 52, 1071–1081. 10.1093/jexbot/52.358.1071 11432923

[B18] Funk J. L. Glenwinkel L. A. Sack L. (2013). Differential allocation to photosynthetic and non-photosynthetic nitrogen fractions among native and invasive species. PLoS ONE 8:e64502. 10.1371/journal.pone.0064502 23700483 PMC3659119

[B19] Gautier L. Cope L. Bolstad B. M. Irizarry R. A. (2004). affy–analysis of Affymetrix genechip data at the probe level. Bioinformatics 20, 307–315. 10.1093/bioinformatics/btg405 14960456

[B20] Giannino D. Nicolodi C. Testone G. Frugis G. Pace E. Santamaria P. (2007). The overexpression of asparagine synthetase A from *E. coli* affects the nitrogen status in leaves of lettuce *(Lactuca sativa* L.) and enhances vegetative growth. Euphytica 162, 11–22. 10.1007/s10681-007-9506-3

[B21] Good A. G. Johnson S. J. De Pauw M. Carroll R. T. Savidov N. Vidmar J. (2007). Engineering nitrogen use efficiency with alanine aminotransferase. Can. J. Bot. 85, 252–262. 10.1139/B07-019

[B22] Good A. G. Shrawat A. K. Muench D. G. (2004). Can less yield more? Is reducing nutrient input into the environment compatible with maintaining crop production? Trends Plant Sci. 9, 597–605. 10.1016/j.tplants.2004.10.008 15564127

[B23] Guo Y. Qiu L. J. (2013). Genome-wide analysis of the Dof transcription factor gene family reveals soybean-specific duplicable and functional characteristics. PLoS ONE 8:e76809. 10.1371/journal.pone.0076809 24098807 PMC3786956

[B24] Habash D. Z. Massiah A. J. Rong H. L. Wallsgrove R. M. Leigh R. A. (2001). The role of cytosolic glutamine synthetase in wheat. Ann. Appl. Biol. 138, 83–89. 10.1111/j.1744-7348.2001.tb00087.x

[B25] Hajdukiewicz P. Svab Z. Maliga P. (1994). The small, versatile pPZP family of Agrobacterium binary vectors for plant transformation. Plant Mol. Biol. 25, 989–994. 10.1007/BF00014672 7919218

[B26] Han M. Okamoto M. Beatty P. H. Rothstein S. J. Good A. G. (2015). The genetics of nitrogen use efficiency in crop plants. Annu. Rev. Genet. 49, 269–289. 10.1146/annurev-genet-112414-055037 26421509

[B27] He X. Qu B. Li W. Zhao X. Teng W. Ma W.. (2015). The nitrate-inducible NAC transcription factor TaNAC2-5A controls nitrate response and increases wheat yield. Plant Physiol. 169, 1991–2005. 10.1104/pp.15.00568 26371233 PMC4634051

[B28] Hoagland D. R. Arnon D. I. (1950). The Water-Culture Method for Growing Plants without Soil, 2nd Edn. Circular, California Agricultural Experiment Station 347, 32.

[B29] Howe A. Sato S. Dweikat I. Fromm M. Clemente T. (2006). Rapid and reproducible Agrobacterium-mediated transformation of sorghum. Plant Cell Rep. 25, 784–791. 10.1007/s00299-005-0081-6 16528567

[B30] Hu L. Li Y. Xu W. Zhang Q. Zhang L. Qi X. (2012). Improvement of the photosynthetic characteristics of transgenic wheat plants by transformation with the maize C_4_ phosphoenolpyruvate carboxylase gene. Plant Breed. 131, 385–391. 10.1111/j.1439-0523.2012.01960.x

[B31] Jeanneau M. Vidal J. Gousset-Dupont A. Lebouteiller B. Hodges M. Gerentes D.. (2002). Manipulating PEPC levels in plants. J. Exp. Bot. 53, 1837–1845. 10.1093/jxb/erf061 12177121

[B32] Koncz C. Schell J. (1986). The promoter of TL-DNA gene 5 controls the tissue-specific expression of chimaeric genes carried by a novel type of Agrobacterium binary vector. Mol. Gen. Genet. 204, 383–396. 10.1007/BF00331014

[B33] Kurai T. Wakayama M. Abiko T. Yanagisawa S. Aoki N. Ohsugi R. (2011). Introduction of the ZmDof1 gene into rice enhances carbon and nitrogen assimilation under low-nitrogen conditions. Plant Biotechnol. J. 9, 826–837. 10.1111/j.1467-7652.2011.00592.x 21624033

[B34] Kushwaha H. Gupta S. Singh V. K. Rastogi S. Yadav D. (2011). Genome wide identification of Dof transcription factor gene family in sorghum and its comparative phylogenetic analysis with rice and Arabidopsis. Mol. Biol. Rep. 38, 5037–5053. 10.1007/s11033-010-0650-9 21161392

[B35] Lijavetzky D. Carbonero P. Vicente-Carbajosa J. (2003). Genome-wide comparative phylogenetic analysis of the rice and Arabidopsis Dof gene families. BMC Evol. Biol. 3:17. 10.1186/1471-2148-3-17 12877745 PMC184357

[B36] Lin W. Hagen E. Fulcher A. Hren M. Cheng Z.-M. (2013). Overexpressing the ZmDof1 gene in Populus does not improve growth and nitrogen assimilation under low-nitrogen conditions. Plant Cell Tissue Organ Cult. 113, 51–61. 10.1007/s11240-012-0250-6

[B37] Liu K. H. Huang C. Y. Tsay Y. F. (1999). CHL1 is a dual-affinity nitrate transporter of Arabidopsis involved in multiple phases of nitrate uptake. Plant Cell 11, 865–874. 10.1105/tpc.11.5.865 10330471 PMC144217

[B38] Luo Z. Q. Clemente T. E. Farrand S. K. (2001). Construction of a derivative of Agrobacterium tumefaciens C58 that does not mutate to tetracycline resistance. Mol. Plant Microbe Interact. 14, 98–103. 10.1094/MPMI.2001.14.1.98 11194879

[B39] Maranville J. W. Madhavan S. (2002). Physiological adaptations for nitrogen use efficiency in sorghum†. Plant Soil 245, 25–34. 10.1023/A:1020660504596

[B40] Markwell J. Osterman J. C. Mitchell J. L. (1995). Calibration of the Minolta SPAD-502 leaf chlorophyll meter. Photosynth. Res. 46, 467–472. 10.1007/BF00032301 24301641

[B41] Martin A. Lee J. Kichey T. Gerentes D. Zivy M. Tatout C.. (2006). Two cytosolic glutamine synthetase isoforms of maize are specifically involved in the control of grain production. Plant Cell 18, 3252–3274. 10.1105/tpc.106.042689 17138698 PMC1693956

[B42] Matsuoka M. Kyozuka J. Shimamoto K. Kano-Murakami Y. (1994). The promoters of two carboxylases in a C_4_ plant (maize) direct cell-specific, light-regulated expression in a C_3_ plant (rice). Plant J. 6, 311–319. 10.1046/j.1365-313X.1994.06030311.x 7920719

[B43] McAllister C. H. Beatty P. H. Good A. G. (2012). Engineering nitrogen use efficient crop plants: the current status. Plant Biotechnol. J. 10, 1011–1025. 10.1111/j.1467-7652.2012.00700.x 22607381

[B44] Meyer Y. Belin C. Delorme-Hinoux V. Reichheld J. P. Riondet C. (2012). Thioredoxin and glutaredoxin systems in plants: molecular mechanisms, crosstalks, and functional significance. Antioxid. Redox Signal. 17, 1124–1160. 10.1089/ars.2011.4327 22531002

[B45] Molina-Rueda J. J. Kirby E. G. (2015). Transgenic poplar expressing the pine GS1a show alterations in nitrogen homeostasis during drought. Plant Physiol. Biochem. 94, 181–190. 10.1016/j.plaphy.2015.06.009 26113157

[B46] Noguero M. Atif R. M. Ochatt S. Thompson R. D. (2013). The role of the DNA-binding One Zinc Finger (DOF) transcription factor family in plants. Plant Sci. 209, 32–45. 10.1016/j.plantsci.2013.03.016 23759101

[B47] Nomura M. Katayama K. Nishimura A. Ishida Y. Ohta S. Komari T. (2000). The promoter of rbcS in a C3 plant (rice) directs organ-specific, light-dependent expression in a C4 plant (maize), but does not confer bundle sheath cell-specific expression. Plant Mol. Biol. 44, 99–106. 10.1023/A:1006461812053 11094984

[B48] O'Leary B. Park J. Plaxton W. C. (2011). The remarkable diversity of plant PEPC (phosphoenolpyruvate carboxylase): recent insights into the physiological functions and post-translational controls of non-photosynthetic PEPCs. Biochem. J. 436, 15–34. 10.1042/BJ20110078 21524275

[B49] Oliveira I. C. Brears T. Knight T. J. Clark A. Coruzzi G. M. (2002). Overexpression of cytosolic glutamine synthetase. Relation to nitrogen, light, and photorespiration. Plant Physiol. 129, 1170–1180. 10.1104/pp.020013 12114571 PMC166511

[B50] Pesquet E. Korolev A. V. Calder G. Lloyd C. W. (2011). Mechanisms for shaping, orienting, positioning and patterning plant secondary cell walls. Plant Signal. Behav. 6, 843–849. 10.4161/psb.6.6.15202 21558816 PMC3218484

[B51] Ritchie M. E. Phipson B. Wu D. Hu Y. Law C. W. Shi W.. (2015). limma powers differential expression analyses for RNA-sequencing and microarray studies. Nucleic Acids Res. 43, e47. 10.1093/nar/gkv007 25605792 PMC4402510

[B52] Sattarzadeh A. Fuller J. Moguel S. Wostrikoff K. Sato S. Covshoff S.. (2010). Transgenic maize lines with cell-type specific expression of fluorescent proteins in plastids. Plant Biotechnol. J. 8, 112–125. 10.1111/j.1467-7652.2009.00463.x 20051034

[B53] Seger M. Gebril S. Tabilona J. Peel A. Sengupta-Gopalan C. (2015). Impact of concurrent overexpression of cytosolic glutamine synthetase (GS1) and sucrose phosphate synthase (SPS) on growth and development in transgenic tobacco. Planta 241, 69–81. 10.1007/s00425-014-2165-4 25213117

[B54] Shaw L. M. McIntyre C. L. Gresshoff P. M. Xue G. P. (2009). Members of the Dof transcription factor family in Triticum aestivum are associated with light-mediated gene regulation. Funct. Integr. Genomics 9, 485–498. 10.1007/s10142-009-0130-2 19578911

[B55] Shrawat A. K. Carroll R. T. DePauw M. Taylor G. J. Good A. G. (2008). Genetic engineering of improved nitrogen use efficiency in rice by the tissue-specific expression of alanine aminotransferase. Plant Biotechnol. J. 6, 722–732. 10.1111/j.1467-7652.2008.00351.x 18510577

[B56] Springer N. M. (2010). Isolation of plant DNA for PCR and genotyping using organic extraction and CTAB. Cold Spring Harb. Protoc. 11, 1559–6095. 10.1101/pdb.prot5515 21041388

[B57] Storey J. D. Tibshirani R. (2003). Statistical significance for genomewide studies. Proc. Natl. Acad. Sci. U.S.A. 100, 9440–9445. 10.1073/pnas.1530509100 12883005 PMC170937

[B58] Uauy C. Distelfeld A. Fahima T. Blechl A. Dubcovsky J. (2006). A NAC Gene regulating senescence improves grain protein, zinc, and iron content in wheat. Science 314, 1298–1301. 10.1126/science.1133649 17124321 PMC4737439

[B59] Wei H. Wang M. L. Moore P. H. Albert H. H. (2003). Comparative expression analysis of two sugarcane polyubiquitin promoters and flanking sequences in transgenic plants. J. Plant Physiol. 160, 1241–1251. 10.1078/0176-1617-01086 14610893

[B60] Xu G. Fan X. Miller A. J. (2012). Plant nitrogen assimilation and use efficiency. Annu. Rev. Plant Biol. 63, 153–182. 10.1146/annurev-arplant-042811-105532 22224450

[B61] Yanagisawa S. (2000). Dof1 and Dof2 transcription factors are associated with expression of multiple genes involved in carbon metabolism in maize. Plant J. 21, 281–288. 10.1046/j.1365-313x.2000.00685.x 10758479

[B62] Yanagisawa S. Akiyama A. Kisaka H. Uchimiya H. Miwa T. (2004). Metabolic engineering with Dof1 transcription factor in plants: improved nitrogen assimilation and growth under low-nitrogen conditions. Proc. Natl. Acad. Sci. U.S.A. 101, 7833–7838. 10.1073/pnas.0402267101 15136740 PMC419692

[B63] Yang X. Tuskan G. A. Cheng M. Z. (2006). Divergence of the Dof gene families in poplar, Arabidopsis, and rice suggests multiple modes of gene evolution after duplication. Plant Physiol. 142, 820–830. 10.1104/pp.106.083642 16980566 PMC1630746

[B64] Zeigler R. S. Mohanty S. (2010). Support for international agricultural research: current status and future challenges. N. Biotechnol. 27, 565–572. 10.1016/j.nbt.2010.08.003 20708721

[B65] Zhu X. G. Long S. P. Ort D. R. (2008). What is the maximum efficiency with which photosynthesis can convert solar energy into biomass? Curr. Opin. Biotechnol. 19, 153–159. 10.1016/j.copbio.2008.02.004 18374559

[B66] Ziemann M. Zachgo S. Bhave M. (2008). The glutaredoxin gene family in wheat functions beyond redox homeostasis regulation, in Proceedings of the 11th International Wheat Genetics Symposium, eds Appels R. Eastwood R. Lagudah E. Langridge P. Mackay M. McIntyre L. Sharp P. (Brisbane, QLD), 585–587.

