# Supplementary material for: Expression of the Maize Dof1 Transcription Factor in Wheat and Sorghum
Source: Front Plant Sci. 2017 Mar 30;8:434. doi: 10.3389/fpls.2017.00434 (PMC5371680; doi:10.3389/fpls.2017.00434)
Supplement: Supplementary file 1 [file DataSheet1.docx]

**Expression of the Maize *Dof1* Transcription Factor in Wheat and Sorghum**

Pamela A. Peña, Truyen Quach, Shirley Sato, Zhengxiang Ge, Natalya Nersesian, Taity Changa, Ismail Dweikat, Madhavan Soundararajan and Tom E. Clemente^*^

^*^Corresponding Author: Tom E. Clemente. Email: [tclemente1@unl.edu](mailto:tclemente1@unl.edu)

**
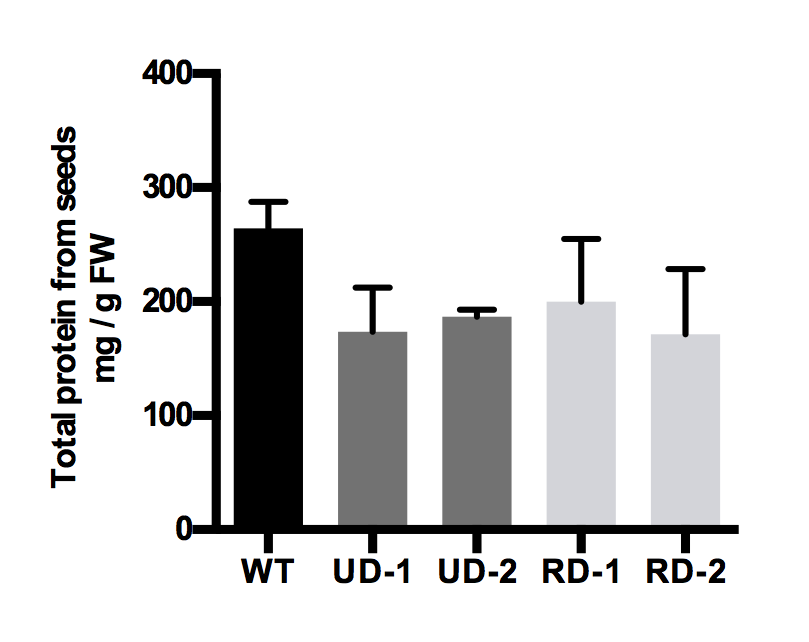
**

**Figure S1. Total protein from seeds of wheat events overexpressing *ZmDof1*.**

Total protein was determined in control plants (WT)-black bars, two UBI4/Dof1 transgenic events (UD-1, UD-2)-grey bars, and two rbcS1/Dof1 transgenic events (RD-1, RD-2)-silver bars. Plants were grown in the field under a nitrogen regime of 95.3 kg/ha residual + applied N. Analysis of variance indicated no significant differences between transgenic events and control plants. Data expressed as mean ± SD (n=3) of protein of bulked seeds from three independent plots.

**Table S1. List of fifty differentially expressed genes that were upregulated according to transcript profiling of the constitutive *ZmDof1* wheat event, UD2, in comparison to control plants. Probe Set ID, annotations according to Rice and Arabidopsis similarities, log2 Fold Change, and adjusted p-value.**

| **Probe Set ID** | **Rice Description** | **Arabidopsis Description** | **logFC** | **adj.P.Val** |
| --- | --- | --- | --- | --- |
| Ta.23165.2.S1_x_at | genomic\|retrotransposon protein, putative, unclassified, expressed | glutaredoxin-related; FUNCTIONS IN: electron carrier activity, protein disulfide oxidoreductase activity; INVOLVED IN: intracellular signaling pathway, cell redox homeostasis | 8.5 | 1.64E-04 |
| Ta.23165.3.S1_x_at | genomic\|retrotransposon protein, putative, unclassified, expressed | glutaredoxin-related; FUNCTIONS IN: electron carrier activity, protein disulfide oxidoreductase activity; INVOLVED IN: intracellular signaling pathway, cell redox homeostasis | 7.5 | 1.64E-04 |
| TaAffx.27775.1.S1_at | cDNA TKL_IRAK_DUF26-lc.24 - DUF26 kinases have homology to DUF26 containing loci, expressed | CRK10 (CYSTEINE-RICH RLK10); ATP binding / kinase/ protein kinase/ protein serine/threonine kinase/ protein tyrosine kinase | 7.3 | 7.08E-04 |
| Ta.23165.1.S1_at | genomic\|retrotransposon protein, putative, unclassified, expressed | glutaredoxin-related; FUNCTIONS IN: electron carrier activity, protein disulfide oxidoreductase activity; INVOLVED IN: intracellular signaling pathway, cell redox homeostasis | 6.5 | 1.55E-04 |
| TaAffx.27177.1.S1_at | cDNA receptor kinase, putative, expressed |  | 5.1 | 3.91E-03 |
| Ta.28339.1.S1_at | cDNA hypothetical protein | unknown protein | 4.6 | 5.59E-04 |
| Ta.27882.3.S1_at |  | Protein of Unknown Function (DUF239) | 4.5 | 4.23E-04 |
| TaAffx.123103.1.S1_at | cDNA expressed protein |  | 4.5 | 1.20E-03 |
| Ta.18694.1.S1_at | cDNA MSP domain containing protein, putative, expressed |  | 4.0 | 2.57E-02 |
| TaAffx.112045.1.S1_x_at | cDNA glutathione S-transferase, putative, expressed | ERD9 (EARLY-RESPONSIVE TO DEHYDRATION 9); glutathione transferase | 3.7 | 5.77E-04 |
| TaAffx.112045.1.S1_at | cDNA glutathione S-transferase, putative, expressed | ERD9 (EARLY-RESPONSIVE TO DEHYDRATION 9); glutathione transferase | 3.6 | 4.97E-03 |
| Ta.8991.1.S1_at | cDNA aldose 1-epimerase, putative, expressed |  | 3.2 | 9.80E-04 |
| Ta.1623.1.S1_at | cDNA serine/arginine repetitive matrix protein 1, putative, expressed | unknown protein | 3.2 | 9.14E-03 |
| TaAffx.63996.1.S1_at | cDNA acetyltransferase, GNAT family, putative, expressed | N-acetyltransferase | 3.2 | 8.62E-04 |
| Ta.13965.1.S1_at | cDNA OsFBX192 - F-box domain containing protein, expressed | F-box family protein (FBW2) | 3.1 | 2.72E-02 |
| Ta.7579.2.A1_at | genomic\|senescence-induced receptor-like serine/threonine-protein kinase precursor, putative, expressed |  | 3.1 | 9.92E-04 |
| Ta.18186.1.A1_at | cDNA LTPL114 - Protease inhibitor/seed storage/LTP family protein precursor, expressed | protease inhibitor/seed storage/lipid transfer protein (LTP) family protein | 3.0 | 8.63E-03 |
| Ta.18619.1.S1_s_at | cDNA inhibitor I family protein, putative, expressed | serine protease inhibitor, potato inhibitor I-type family protein | 3.0 | 2.72E-02 |
| TaAffx.7104.1.S1_at | cDNA DNA-directed RNA polymerase III subunit RPC8, putative, expressed | RNA polymerase Rpb7 N-terminal domain-containing protein | 2.9 | 2.44E-02 |
| Ta.22614.1.S1_at |  | serine protease inhibitor, potato inhibitor I-type family protein | 2.7 | 2.06E-02 |
| Ta.5107.1.A1_at | cDNA transcription factor X1, putative, expressed | XH/XS domain-containing protein / XS zinc finger domain-containing protein | 2.6 | 2.72E-02 |
| TaAffx.32247.1.S1_at | cDNA hypothetical protein | unknown protein | 2.6 | 1.08E-02 |
| Ta.26120.1.S1_x_at | cDNA retrotransposon protein, putative, unclassified | protein kinase family protein | 2.5 | 8.25E-03 |
| Ta.9080.1.A1_s_at | cDNA ATMAP70 protein, putative, expressed |  | 2.5 | 4.96E-03 |
| Ta.12046.1.A1_at | genomic\|ubiquitin family protein, putative, expressed |  | 2.5 | 4.96E-03 |
| Ta.25492.1.S1_at | cDNA oxidoreductase, aldo/keto reductase family protein, putative, expressed | ATB2; oxidoreductase | 2.5 | 3.51E-02 |
| TaAffx.7104.1.S1_x_at | cDNA DNA-directed RNA polymerase III subunit RPC8, putative, expressed | RNA polymerase Rpb7 N-terminal domain-containing protein | 2.4 | 2.49E-02 |
| Ta.4748.1.S1_at | cDNA nucleoside diphosphate kinase, putative, expressed | NDPK1; ATP binding / nucleoside diphosphate kinase | 2.2 | 2.22E-02 |
| TaAffx.40923.2.S1_at | cDNA G-patch domain containing protein, expressed | D111/G-patch domain-containing protein | 2.1 | 4.83E-03 |
| Ta.26120.1.S1_at | cDNA retrotransposon protein, putative, unclassified | protein kinase family protein | 2.1 | 1.01E-02 |
| TaAffx.38265.5.S1_at | cDNA MATH domain containing protein |  | 2.1 | 4.62E-02 |
| TaAffx.82189.1.S1_at | protein retrotransposon protein, putative, unclassified, expressed |  | 2.1 | 4.73E-02 |
| Ta.26911.1.S1_at |  | peptidase M48 family protein | 2.1 | 1.61E-02 |
| Ta.26922.1.S1_at | cDNA oxidoreductase, aldo/keto reductase family protein, putative, expressed | aldo/keto reductase family protein | 2.0 | 3.93E-02 |
| Ta.116.1.S1_at | cDNA IN2-1 protein, putative, expressed | GSTL2 | 2.0 | 4.12E-02 |
| Ta.22638.1.A1_at | cDNA resistance-related receptor-like kinase, putative | PR5K; kinase/ transmembrane receptor protein serine/threonine kinase | 2.0 | 5.06E-03 |
| Ta.2929.1.S1_a_at | cDNA deoxyuridine 5-triphosphate nucleotidohydrolase, putative, expressed | deoxyuridine 5'-triphosphate nucleotidohydrolase family | 2.0 | 5.42E-03 |
| TaAffx.119976.1.A1_at | cDNA formin-like protein 3 precursor, putative, expressed |  | 1.9 | 1.82E-02 |
| Ta.14076.1.S1_at | cDNA conserved hypothetical protein |  | 1.9 | 2.72E-02 |
| TaAffx.122846.3.S1_s_at | cDNA OsSCP19 - Putative Serine Carboxypeptidase homologue, expressed | scpl2 (serine carboxypeptidase-like 2); serine-type carboxypeptidase | 1.9 | 2.72E-02 |
| TaAffx.6564.1.S1_at | cDNA cytochrome P450, putative, expressed | CYP71A25; electron carrier/ heme binding / iron ion binding / monooxygenase/ oxygen binding | 1.9 | 3.51E-02 |
| Ta.7207.1.A1_at | cDNA ankyrin repeat domain-containing protein, putative, expressed | ankyrin repeat family protein | 1.9 | 9.14E-03 |
| Ta.28518.1.S1_at | cDNA zinc finger, C3HC4 type domain containing protein, expressed |  | 1.8 | 2.72E-02 |
| Ta.941.1.A1_at | cDNA cysteine-rich receptor-like protein kinase 7 precursor, putative, expressed | protein kinase family protein | 1.8 | 2.29E-02 |
| Ta.23307.3.S1_a_at | cDNA expressed protein |  | 1.8 | 2.72E-02 |
| TaAffx.12718.1.A1_at | cDNA hypothetical protein |  | 1.7 | 1.72E-02 |
| TaAffx.129414.2.S1_at | cDNA receptor-like kinase ARK1AS, putative, expressed | protein kinase family protein | 1.7 | 8.96E-03 |
| Ta.8165.2.S1_a_at | cDNA cysteine-rich receptor-like protein kinase 21 precursor, putative, expressed | protein kinase family protein | 1.7 | 1.58E-02 |
| Ta.8814.1.A1_at | cDNA xylanase inhibitor, putative, expressed | extracellular dermal glycoprotein, putative / EDGP, putative | 1.7 | 2.37E-02 |
| Ta.21091.1.S1_s_at | cDNA conserved hypothetical protein |  | 1.6 | 4.81E-02 |

**Table S2. List of fifty differentially expressed genes that were downregulated according to transcript profiling of the constitutive *ZmDof1* wheat event, UD2, in comparison to control plants. Probe Set ID, annotations according to Rice and Arabidopsis similarities, log2 Fold Change, and adjusted p-value.**

| **Probe Set ID** | **Rice Description** | **Arabidopsis Description** | **logFC** | **adj.P.Val** |
| --- | --- | --- | --- | --- |
| Ta.611.1.A1_at |  | ULP1B (UB-LIKE PROTEASE 1B); cysteine-type peptidase | -7.9 | 5.59E-04 |
| Ta.28123.1.S1_at | cDNA ribosomal protein L28 protein, putative, expressed | 50S ribosomal protein L28, chloroplast (CL28) | -6.7 | 1.55E-04 |
| Ta.7158.1.S1_at |  | Arabidopsis thaliana TCP family transcription factor. Regulated by miR319. Involved in heterchronic regulation of leaf differentiation. | -6.0 | 3.06E-03 |
| TaAffx.58902.1.S1_x_at | Putative NADH-ubiquinone oxireductase |  | -5.0 | 2.50E-04 |
| TaAffx.120564.1.A1_at | cDNA expressed protein |  | -4.5 | 7.32E-03 |
| TaAffx.12662.1.S1_at | cDNA phosphoenolpyruvate carboxylase, putative, expressed | ATPPC1 (PHOSPHOENOLPYRUVATE CARBOXYLASE 1); catalytic/ phosphoenolpyruvate carboxylase | -4.3 | 4.08E-04 |
| Ta.28354.4.S1_at | cDNA glutathione S-transferase, putative, expressed | ERD9 (EARLY-RESPONSIVE TO DEHYDRATION 9); glutathione transferase | -4.2 | 1.85E-03 |
| Ta.6425.1.A1_at | cDNA expressed protein |  | -3.5 | 1.56E-02 |
| Ta.8601.1.S1_at | cDNA pentatricopeptide, putative, expressed | pentatricopeptide (PPR) repeat-containing protein | -3.3 | 1.53E-02 |
| TaAffx.81369.1.S1_at | cDNA protein kinase domain containing protein, expressed | SD2-5 (S-DOMAIN-2 5); carbohydrate binding / kinase/ protein kinase | -3.2 | 8.62E-04 |
| Ta.21557.1.A1_at | cDNA senescence-associated protein, putative, expressed | senescence/dehydration-associated protein-related | -2.8 | 2.70E-03 |
| Ta.13670.3.S1_at | cDNA ORM1, putative, expressed | ORMDL family protein | -2.5 | 2.44E-02 |
| Ta.22178.1.A1_at |  | transferase family protein | -2.5 | 7.89E-03 |
| Ta.23013.3.S1_s_at | cDNA expressed protein | MT2B (METALLOTHIONEIN 2B); copper ion binding | -2.5 | 1.06E-02 |
| TaAffx.53898.1.S1_at | cDNA receptor-like kinase ARK1AS, putative, expressed |  | -2.4 | 9.91E-03 |
| Ta.28611.1.S1_at | cDNA osmotin, putative, expressed |  | -2.3 | 1.44E-02 |
| Ta.608.3.S1_at | cDNA DNA-directed RNA polymerase II subunit RPB4, putative, expressed | NRPB4; DNA-directed RNA polymerase | -2.1 | 2.62E-02 |
| Ta.10531.1.S1_at | cDNA serine/arginine repetitive matrix protein 1, putative, expressed |  | -2.1 | 2.37E-02 |
| Ta.30903.1.A1_at | cDNA membrane associated DUF588 domain containing protein, putative, expressed | integral membrane family protein | -2.1 | 4.40E-02 |
| Ta.21397.1.S1_at | cDNA tropinone reductase 2, putative | tropinone reductase, putative / tropine dehydrogenase, putative | -2.0 | 9.81E-03 |
| Ta.10531.2.S1_a_at | cDNA serine/arginine repetitive matrix protein 1, putative, expressed |  | -2.0 | 8.29E-03 |
| Ta.22359.1.S1_at |  | catalytic/ protein binding / serine-type endopeptidase/ serine-type peptidase | -2.0 | 1.82E-02 |
| Ta.25417.1.S1_at | cDNA retrotransposon protein, putative, unclassified, expressed | CW7 | -1.9 | 3.46E-02 |
| TaAffx.72768.2.S1_at | cDNA protein kinase domain containing protein, expressed |  | -1.8 | 3.91E-02 |
| Ta.4940.1.A1_at |  | actin binding | -1.8 | 3.93E-02 |
| Ta.27916.1.A1_s_at | cDNA transmembrane amino acid transporter protein, putative, expressed | amino acid transporter family protein | -1.6 | 2.73E-02 |
| TaAffx.66666.1.A1_at | cDNA phosphatidylinositol-4-phosphate 5-Kinase, putative, expressed | phosphatidylinositol-4-phosphate 5-kinase family protein | -1.6 | 4.94E-02 |
| Ta.27916.1.A1_x_at | cDNA transmembrane amino acid transporter protein, putative, expressed | amino acid transporter family protein | -1.6 | 3.46E-02 |
| TaAffx.8804.3.S1_s_at | cDNA aquaporin protein, putative, expressed | PIP2B (PLASMA MEMBRANE INTRINSIC PROTEIN 2); water channel | -1.6 | 4.61E-02 |
| Ta.18888.1.S1_at | cDNA hydrolase, NUDIX family, domain containing protein, expressed | atnudt12 (Arabidopsis thaliana Nudix hydrolase homolog 12); hydrolase | -1.5 | 2.72E-02 |
| TaAffx.110726.1.S1_s_at | cDNA aspartic proteinase nepenthesin-2 precursor, putative, expressed | chloroplast nucleoid DNA-binding protein, putative | -1.5 | 3.77E-02 |
| TaAffx.54367.1.S1_at | cDNA CBS domain containing membrane protein, putative, expressed |  | -1.5 | 2.72E-02 |
| Ta.9751.1.A1_at | cDNA ELMO/CED-12 family protein, putative, expressed | ATATH8; transporter | -1.4 | 1.73E-02 |
| Ta.11218.1.A1_at | cDNA transposon protein, putative, CACTA, En/Spm sub-class, expressed |  | -1.4 | 1.53E-02 |
| Ta.28750.1.S1_x_at | cDNA photosystem II 10 kDa polypeptide, chloroplast precursor, putative, expressed | PSBR (photosystem II subunit R) | -1.4 | 2.57E-02 |
| Ta.30321.1.S1_at | cDNA abscisic stress-ripening, putative, expressed | protein kinase family protein | -1.4 | 1.72E-02 |
| Ta.8359.1.S1_s_at | cDNA osmotin, putative, expressed | osmotin-like protein, putative | -1.4 | 2.07E-02 |
| Ta.28750.1.S1_at | cDNA photosystem II 10 kDa polypeptide, chloroplast precursor, putative, expressed | PSBR (photosystem II subunit R) | -1.4 | 2.44E-02 |
| Ta.3979.1.S1_at | cDNA mitochondrial carrier protein, putative, expressed | BOU (A BOUT DE SOUFFLE); binding / transporter | -1.4 | 4.56E-02 |
| Ta.23348.1.A1_x_at | cDNA aspartic proteinase nepenthesin-2 precursor, putative, expressed | chloroplast nucleoid DNA-binding protein, putative | -1.4 | 3.75E-02 |
| Ta.23348.1.A1_at | cDNA aspartic proteinase nepenthesin-2 precursor, putative, expressed | chloroplast nucleoid DNA-binding protein, putative | -1.4 | 4.84E-02 |
| TaAffx.30205.2.S1_s_at | cDNA AMP-binding domain containing protein, expressed | 4CL2 (4-COUMARATE:COA LIGASE 2); 4-coumarate-CoA ligase | -1.3 | 4.56E-02 |
| Ta.20870.1.S1_at | cDNA haemolysin-III, putative, expressed | HHP1 (HEPTAHELICAL TRANSMEMBRANE PROTEIN1); receptor | -1.3 | 2.94E-02 |
| Ta.23366.2.S1_at | cDNA peroxidase precursor, putative, expressed | peroxidase 12 (PER12) (P12) (PRXR6) | -1.3 | 2.75E-02 |
| Ta.23366.2.S1_x_at | cDNA peroxidase precursor, putative, expressed | peroxidase 12 (PER12) (P12) (PRXR6) | -1.3 | 2.72E-02 |
| Ta.25381.1.S1_s_at | cDNA aspartic proteinase, putative, expressed | aspartyl protease family protein | -1.3 | 4.63E-02 |
| TaAffx.56014.2.S1_x_at | cDNA aspartic proteinase nepenthesin-2 precursor, putative, expressed | aspartyl protease family protein | -1.3 | 2.47E-02 |
| TaAffx.129824.10.S1_at | cDNA DNA-directed RNA polymerase subunit alpha, putative | RNA polymerase alpha subunit | -1.2 | 2.72E-02 |
| Ta.10216.1.A1_at | cDNA aspartic proteinase nepenthesin-2 precursor, putative, expressed | chloroplast nucleoid DNA-binding protein, putative | -1.2 | 3.96E-02 |
| TaAffx.29399.1.S1_at | cDNA transmembrane amino acid transporter protein, putative, expressed | amino acid transporter family protein | -1.2 | 4.94E-02 |

**Table S3. Primers utilized for validation of microarray data using qRT-PCR.**
